# Apolipoprotein D neofunctionalization couples lipid allocation to wing evolution

**DOI:** 10.1038/s44318-026-00821-0

**Published:** 2026-06-03

**Authors:** Yuxin Huang, Yinghui Li, Shunze Jia, Yanting Liang, Qingyu Lu, Wenhui Jing, Huabing Wang

**Affiliations:** College of Animal Sciences, Zhejiang University, Hangzhou, China

**Keywords:** Evolution & Ecology, Metabolism

## Abstract

The origin of insect wings marked a pivotal evolutionary innovation that enabled their extraordinary ecological success, yet the metabolic mechanisms sustaining this transition remain elusive. Here, we identify apolipoprotein D2 (ApoD2)—a neofunctionalized paralog of apolipoprotein D—as a key metabolic regulator that spatially coordinates lipid allocation during lepidopteran wing development. Comparative phylogenomics across 791 metazoan genomes revealed that ApoD2 emerged as a lepidopteran duplicate exhibiting sustained, wing-enriched expression across developmental stages. Using *Bombyx mori* as a model, we show that ApoD2 is indispensable for wing morphogenesis, coupling lipid compartmentalization to local energetic demands. Loss of ApoD2 disrupts mitochondrial bioenergetics and fatty acid oxidation, leading to depletion of wing muscle cells. Lipidomic profiling further revealed that ApoD2 deficiency causes systemic lipid misallocation—characterized by hemolymph fatty acid accumulation and depletion of diglycerides and morphogenic lipids in wings, triggering AMPK-dependent autophagy. Mechanistically, duplicated ApoD2 integrates systemic lipid transport with organ-specific energy deployment, linking metabolic rewiring to morphological innovation. Together, these findings reveal how the neofunctionalization of a metabolic regulator resolved evolutionary trade-offs between energy efficiency and structural complexity, illuminating a general principle by which metabolic innovation drives the evolution of complex traits in insects.

## Synopsis

The evolution of insect wings required profound metabolic innovation. This study identifies the lepidopteran paralog ApoD2 as a spatial regulator that rewires lipid metabolism to fuel wing morphogenesis by enabling compartmentalized lipid trafficking and energy homeostasis.

- Lepidopteran paralog ApoD2 is a spatial regulator that rewires lipid metabolism to fuel wing morphogenesis.
- Loss of ApoD2 disrupts mitochondrial bioenergetics and fatty acid oxidation, leading to depletion of wing muscle cells.
- ApoD2 deficiency triggers hemolymph fatty acid accumulation and concurrent depletion of wing diglycerides and morphogenic lipids.
- ApoD2 underwent neofunctionalization to mediate spatially resolved lipid allocation during wing development.

## Abstract (web summary)

Metabolic adaptation via a lepidopteran paralog gene drives the evolution of insect complex wing traits.

## Graphical abstract

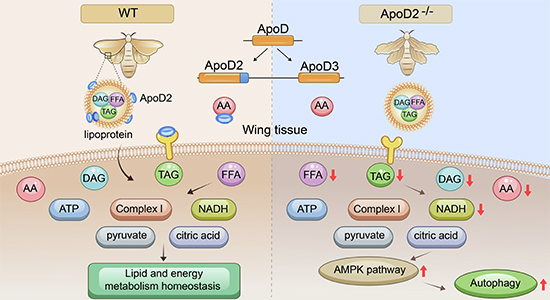

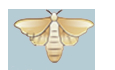

## Introduction

The emergence of novel morphological traits has repeatedly reshaped animal evolution, yet such innovations ultimately depend on metabolic systems that sustain developmental and physiological complexity. Metabolic pathways not only provide energy but also shape organ function and evolutionary potential. Understanding how genomic changes rewire metabolism to enable new morphologies remains a central challenge in evolutionary biology. Over deep time, selective pressures have sculpted genome architecture, giving rise to lineage-specific metabolic capacities (Fondi et al, 2009; Pontzer et al, 2016). However, the genomic mechanisms that couple metabolic innovation with morphological diversification—particularly at the organ level—remain poorly defined.

The evolutionary success of insects over the past ~400 million years is closely linked to the origin and diversification of wings (Wootton and Kukalová-Peck, 2000). While the biomechanics and developmental genetic programs underlying wing morphogenesis have been extensively characterized, the metabolic strategies that sustain wing growth and enable flight performance remain less well understood. Lipid metabolism is particularly central in this context, providing both essential structural components and high-energy substrates required for sustained flight (Panáková et al, 2005). However, how lipid allocation is coordinated across tissues during wing development, and how such systemic metabolic regulation evolved, remain poorly understood.

Apolipoproteins, which mediate the transport of hydrophobic lipids, play essential roles in maintaining cellular and systemic energy balance (Flower, 1996; Sanchez and Ganfornina, 2021). Among them, Apolipoprotein D (ApoD) is a multifunctional lipid-binding protein implicated in oxidative stress resistance, nutrient signaling, and lifespan regulation (Zhou et al, 2018). Insects have diversified their *ApoD* gene repertoire through lineage-specific duplications. For instance, *Drosophila melanogaster* encodes two paralogs—neural lazarillo (Nlaz) and glial lazarillo (Glaz)—that regulate stress tolerance and metabolic homeostasis (Ruiz et al, 2014; Ruiz et al, 2011). Lepidopterans harbor three ApoD paralogs. Notably, ApoD2, a lepidopteran duplicate, is highly expressed in developing wings and features a distinctive C-terminal extension (Jia et al, 2024). However, the molecular and evolutionary mechanisms by which ApoD diversification supports wing-specific metabolism remain unknown.

In this study, we conducted a comprehensive evolutionary analysis of the *ApoD* gene family across 791 animal genomes, with a particular focus on insect lineages. By integrating single-cell transcriptomics, lipidomics, and targeted metabolomics in the lepidopteran model *Bombyx mori*, we uncovered a coordinated mechanism whereby gene duplication and neofunctionalization of ApoD paralogs drive tissue-specific metabolic reprogramming during wing development. Our findings show that lepidopteran duplicated ApoD orchestrate lipid partitioning and energy metabolism through a multilayered regulatory network, aligning systemic lipid homeostasis with localized morphogenetic cues. These findings establish a conceptual framework in which paralog diversification acts as an evolutionary strategy to resolve organ-level trade-offs between morphological complexity and metabolic efficiency.

## Results

### ApoD families underwent convergent expansions in insects and Actinopteri

**Figure 1:**
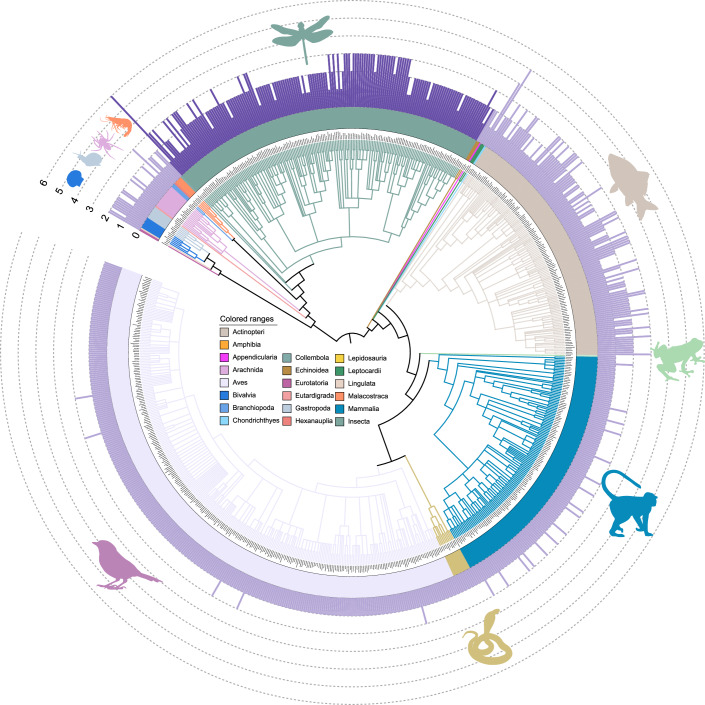
Quantitative analysis of *ApoD* gene distribution across 791 animal species. A maximum likelihood phylogenetic tree of 791 metazoan species reveals 1154 *ApoD* genes. Purple stripes indicate the number of *ApoD* homologs, with each concentric circle increment representing one additional gene. Distinct colors correspond to different animal classes. Source data are available online for this figure.

To trace the evolutionary history of Apolipoprotein D—a conserved lipid-binding protein involved in energy metabolism, oxidative stress defense, and lifespan regulation, we performed a comprehensive phylogenomic survey across 791 animal genomes spanning 20 major lineages (Fig. 1; Datasets EV1 and 2). In mammals, birds, and reptiles, *ApoD* is typically represented by a single gene, reflecting strong evolutionary constraint. In contrast, both ray-finned fishes (Actinopteri) and insects exhibit extensive *ApoD* duplication, suggesting recurrent selective pressures for metabolic specialization. Among Actinopteri, ~23% of species possess three ApoD copies, while nearly 90% of insects retain two or more. These independent expansions across distant taxa point to convergent molecular strategies for enhancing lipid transport and energy regulation in metabolically demanding systems.

**Figure 2:**
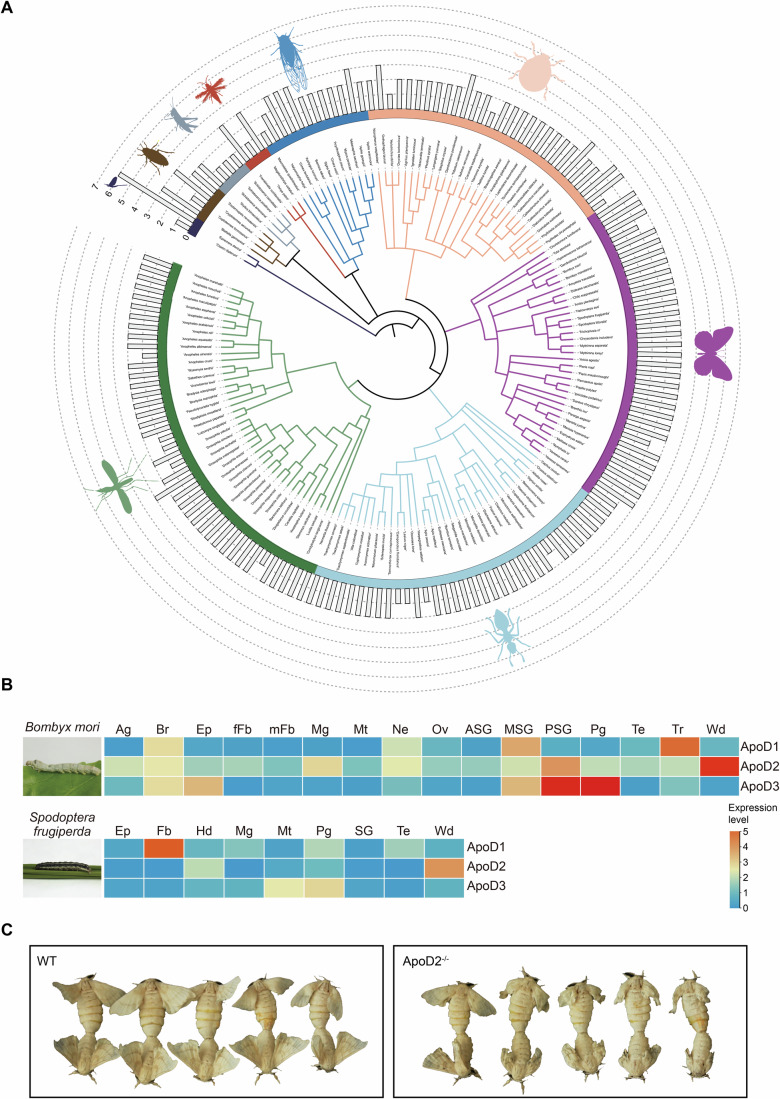
Comparative analysis of *ApoD* genes within Insecta. (**A**) A maximum likelihood phylogenetic tree of *ApoD* genes across nine insect orders, displaying 355 *ApoD* genes. Bars represent the number of *ApoD* genes, with each concentric circle increment indicating one additional gene. Colors denote different insect orders. (**B**) Spatiotemporal expression profiles of three *ApoD* genes in two lepidopteran species. Tissues and organs include: Br brain, Hd head, Ep epidermis, Ne nerve, mFb male fat body, fFb female fat body, Fb fat body, Ag anterior gut, Mg, midgut Pg posterior gut, ASG anterior silk gland, MSG middle silk gland, PSG posterior silk gland, SG silk gland, Mt malpighian tubule, Ov ovary, Te testis, Tr trachea, Wd wing disc. (**C**) Wing phenotypes of wild-type and *ApoD2*^−/−^ mutant silkworm. Source data are available online for this figure.

**Figure EV1:**
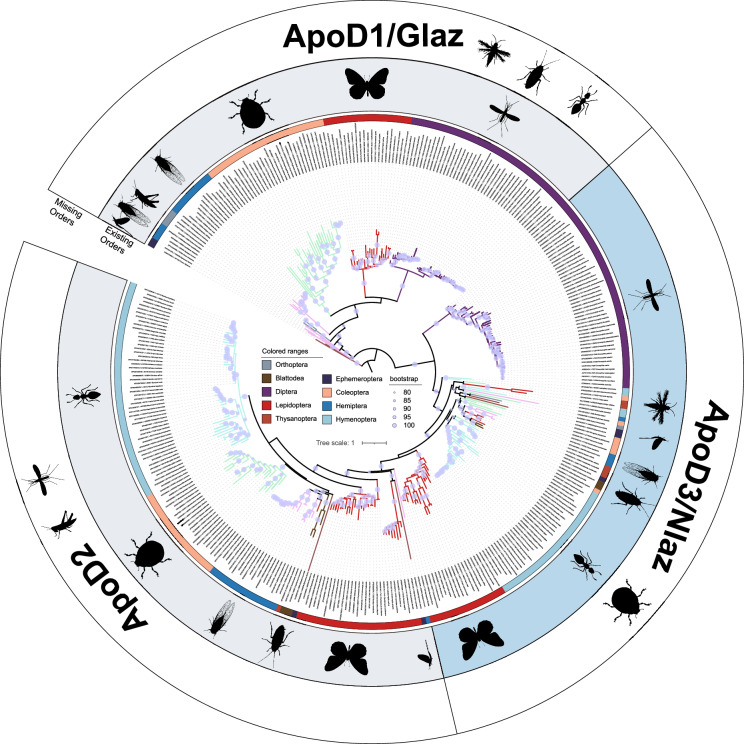
Phylogenetic tree of *ApoD* genes in the Insecta section. The colors of the inner circles represent different insect orders, the middle circles represent insect orders with *ApoD* gene subfamilies, and the outermost circles represent different *ApoD* gene subfamilies and insect orders lacking the genes of the subfamily.

Within insects, *ApoD* copy number varies markedly among orders (Fig. 2A; Dataset EV3). Coleoptera, Hymenoptera, Diptera, and Hemiptera typically harbor one or two *ApoD* genes, whereas Lepidoptera display consistent expansion, maintaining three distinct subfamilies: *ApoD1*, *ApoD2*, and *ApoD3*. Phylogenetic reconstruction revealed that *ApoD1* and *ApoD2* are retained in Coleoptera, *ApoD2* and *ApoD3* in Hymenoptera, and *ApoD1* and *ApoD3* in Diptera, highlighting modular subfamily retention across orders (Fig. EV1). By contrast, lepidopteran genomes uniquely preserve all three subfamilies, with *ApoD2* and *ApoD3* arising from a tandem duplication event maintained in conserved syntenic blocks across species (Jia et al, 2024). Together, these results uncover a clear phylogenetic asymmetry in *ApoD* evolution: while most vertebrates maintain a single conserved gene, insects—particularly Lepidoptera—have undergone repeated duplication and retention events.

### ApoD2 neofunctionalization is required for lepidopteran wing morphogenesis

The three lepidopteran *ApoD* paralogs display strikingly distinct spatial expression patterns. In *B. mori* and *Spodoptera frugiperda*, *ApoD1* is mainly expressed in metabolically active tissues such as the trachea, midgut, and fat body, while *ApoD3* predominates in the posterior silk glands and hindgut. By contrast, the duplicated *ApoD2* shows marked enrichment in the developing wing disc of both species (Fig. 2B), suggesting a specialized role in wing development.

**Figure EV2:**
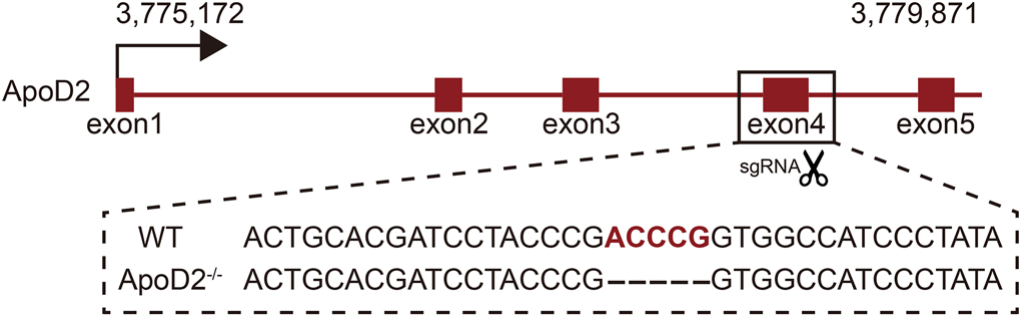
Schematic diagram of *ApoD2* gene knockout. The *ApoD2* gene structure showing five exons is illustrated, with the sgRNA target site located in exon 4. The sequence ACCCG is deleted in the *ApoD2*^−/−^ mutant, resulting in a 5-bp deletion.

To test this hypothesis, we previously generated *ApoD2*-null mutants in *B. mori* using CRISPR/Cas9-mediated genome editing (Fig. EV2, Jia et al, 2024). The loss of *ApoD2* resulted in a severe wing-curling phenotype affecting both forewings and hindwings, irrespective of sex (Fig. 2C). These results establish ApoD2 as an essential determinant of wing morphogenesis, and its duplication likely enabled the evolution of tissue-specific lipid regulatory networks.

### Single-cell transcriptomics reveals mitochondrial collapse and metabolic stress in ApoD2-deficient wings

**Figure 3:**
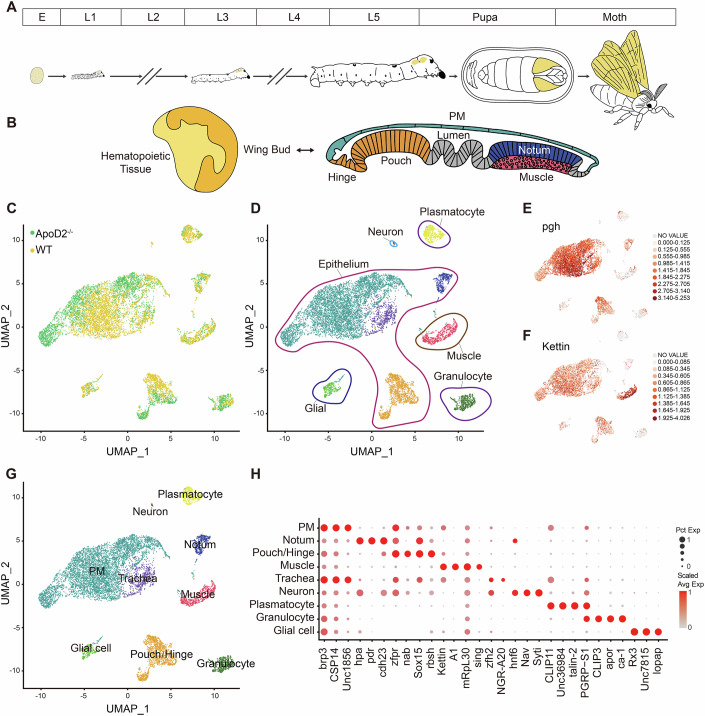
Wing cell atlas of wild-type and *ApoD2*^−^^/−^ mutants at the P3 stage. (**A**) Developmental timeline of the silkworm: E, embryo; L1–L5, larval stages; Pupa, pupal stage; Moth, adult stage. (**B**) Schematic of the silkworm wing bud. Epithelial cells in the wing bud differentiate into the wing blade, hinge, and most of the thoracic dorsum in the adult moth. Pouch/Hinge: develops into the hinge connecting the wing to the body; Notum: forms the sclerotized dorsal structure; PM, peripodial membrane; Muscle: flight muscle. (**C**) UMAP visualization of the integrated single-cell dataset, with cells colored by major cell type: *ApoD2*^−/−^ mutant cells (green) and wild-type cells (yellow). (**D**) Major cell types identified in silkworm wing tissue. (**E**, **F**) Epithelial and muscle cells are distinguished by the expression of protein grainyhead (pgh) and kettin protein (Kettin), respectively. (**G**) UMAP of wing cells after cell type segmentation. (**H**) Dot plot showing marker gene expression used for cell type annotation. Dot size represents the percentage of cells expressing the gene within a cell type, and color intensity indicates the average expression level. Source data are available online for this figure.

**Figure EV3:**
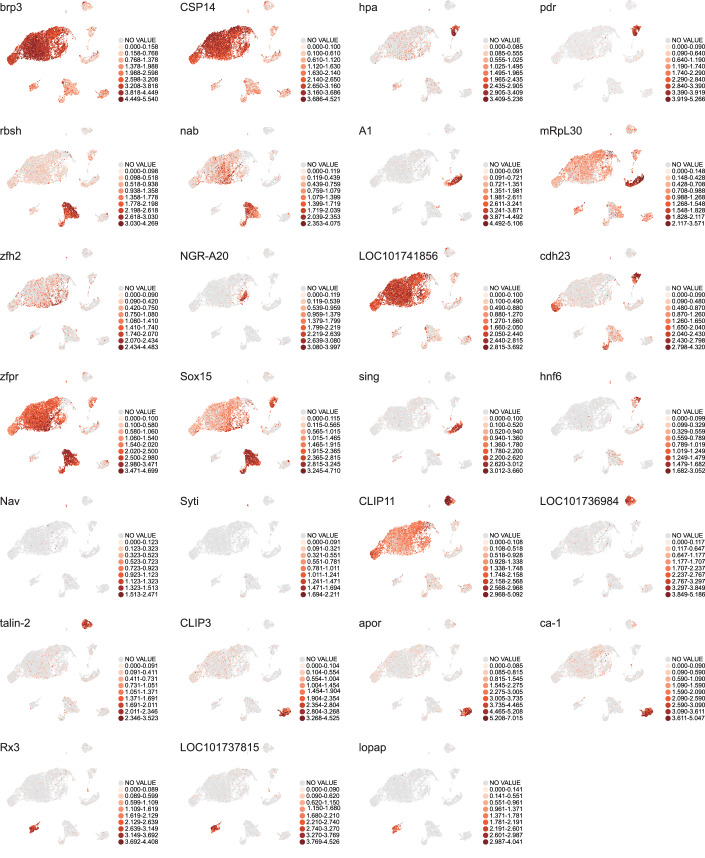
Expression of marker genes in each cell type. Epithelial cells were identified using the marker gene *pgh* (Deng et al, 2021; Ma et al, 2017), while muscle cells were identified by the expression of *Kettin* (Bullard et al, 2002; Lakey et al, 1993), *A1* (Timson et al, 1998; Wawro et al, 2022), *mRpL30* (Cheong et al, 2020; Rai et al, 2024), and *sing* (Brunetti et al, 2015). Tracheal cells were labeled with *zfh2* (Perea et al, 2013; Terriente et al, 2008) and *NGR-A20* (Grundemar, 1997; Wu et al, 2024), while neurons were labeled with *hnf6* (Audouard et al, 2012; Francius and Clotman, 2010), *Nav* (Feng et al, 1995; Planells-Cases et al, 2000), and *Syti* (Liu et al, 2009; Ro et al, 2010). Glial cells were labeled with *lopap* (Fritzen et al, 2005; Reis et al, 1999) and *Rx3* (Kraft et al, 2016; Zagozewski et al, 2014), and immune-related cell types included plasmatocytes labeled with *CLIP11* (Kanost and Jiang, 2015) and *talin-2* (von Essen et al, 2016) and granulocytes labeled with *CLIP3* (Vaseeharan et al, 2011; Zhang et al, 2018), *apor* (Brankatschk and Eaton, 2010; Canavoso and Rubiolo, 1994), and *ca-1* (Pipoly and Crouch, 1987).

**Figure EV4:**
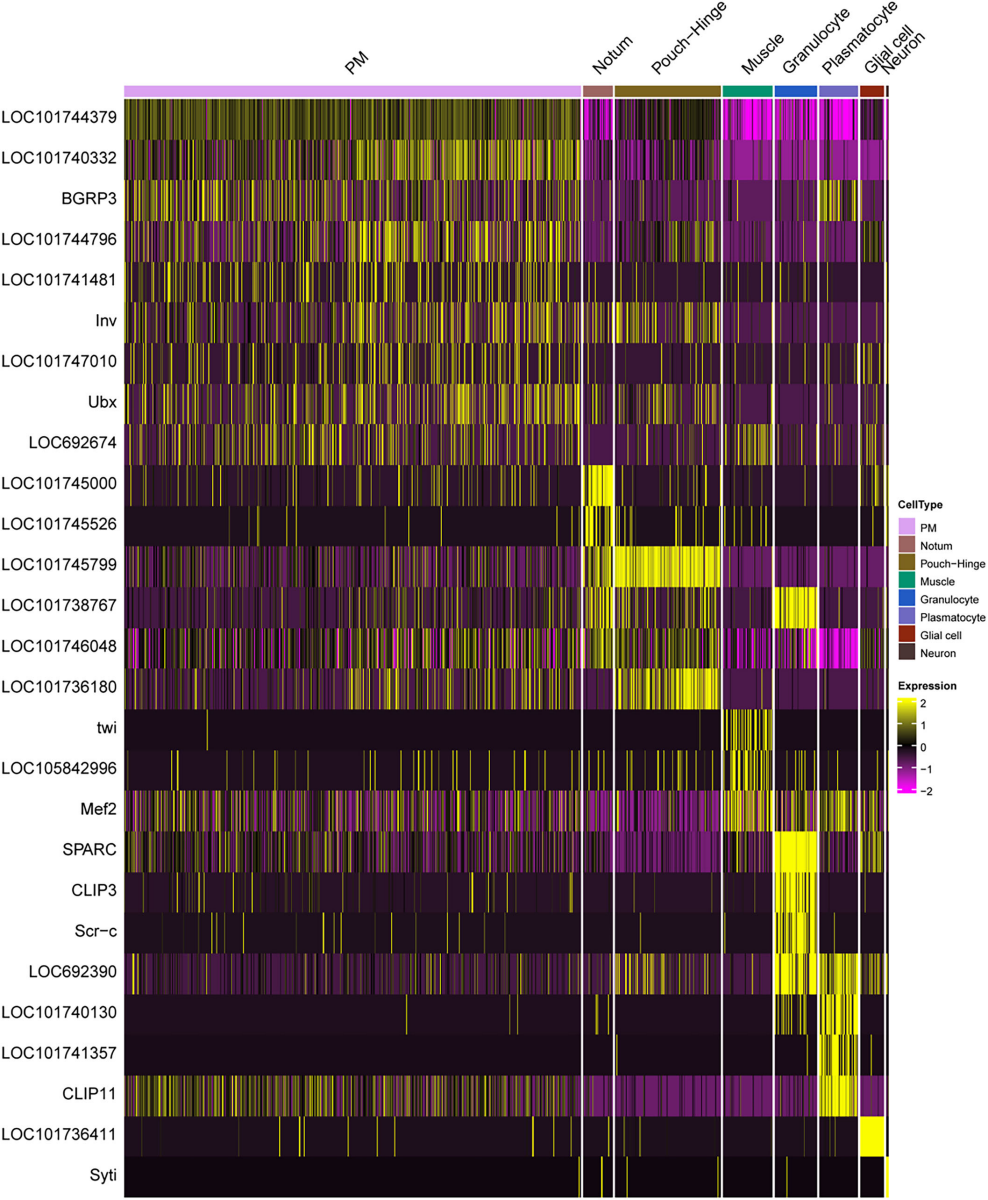
Heatmap of the proportional expression values of the top ten most upregulated genes in different cell types of silkworm wing tissue. Heatmap of the proportional expression values of the top upregulated genes in different cell types of silkworm wing tissue. Each column represents a single cell, and each row represents a gene. Colors indicate scaled expression levels, ranging from high (yellow) to low (purple). Cell types are indicated by colored bars at the top of the heatmap.

**Figure EV5:**
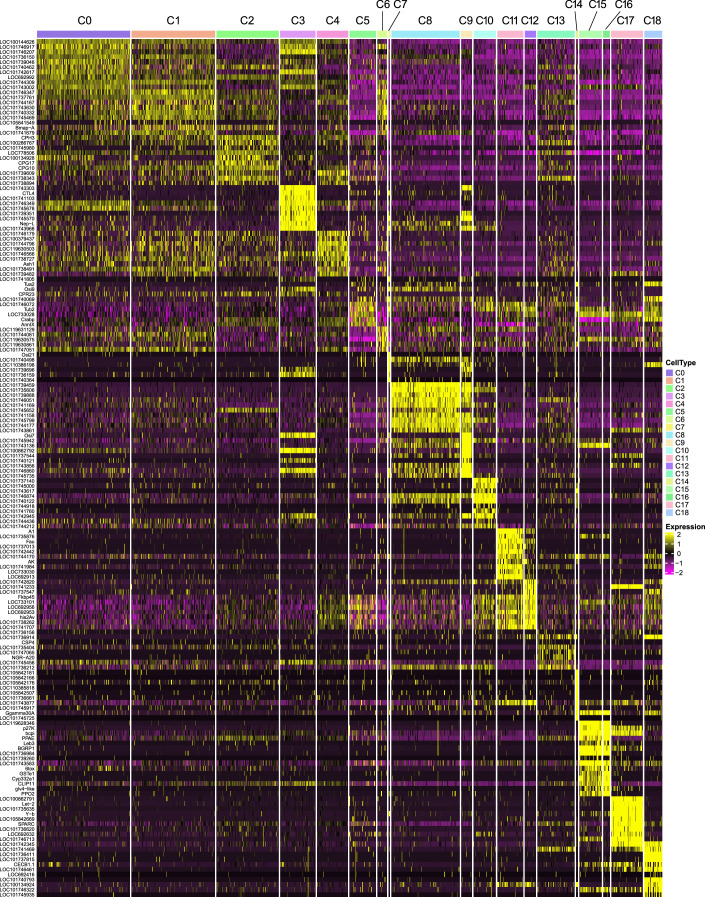
Heatmap of the proportional expression values of the top ten most upregulated genes. Subclassification of epithelial cells was achieved using markers: *brp3* and *CSP14* for peripodial membrane (PM); *hpa* (Gómez-Skarmeta and Modolell, 1996), *pdr*, and *cdh23* for notum; *nab*, *zfpr* (Staehling-Hampton et al, 1995; Wu et al, 2019), *rbsh* (Klein et al, 2000), *Sox15* (Song et al, 2019) for pouch/hinge.

To investigate how ApoD2 deficiency affects cellular programs during wing morphogenesis, we performed single-cell RNA sequencing (scRNA-seq) of pupal wings from *B. mori* wild-type (WT) and *ApoD2*^−/−^ mutants (Figs. 3A–C and EV3–5). Transcriptomic profiling of over 10,000 high-quality cells revealed six major cell types, including epithelial, muscle, tracheal, neuronal, glial, and immune populations (Fig. 3D–F; Dataset EV4). Further subclassification of epithelial cells distinguished the pouch/hinge, notum, and peripodial membrane domains—developmental regions essential for wing articulation and flight control (Everetts et al, 2021; Lecuit and Cohen, 1998) (Fig. 3G,H).

**Figure 4:**
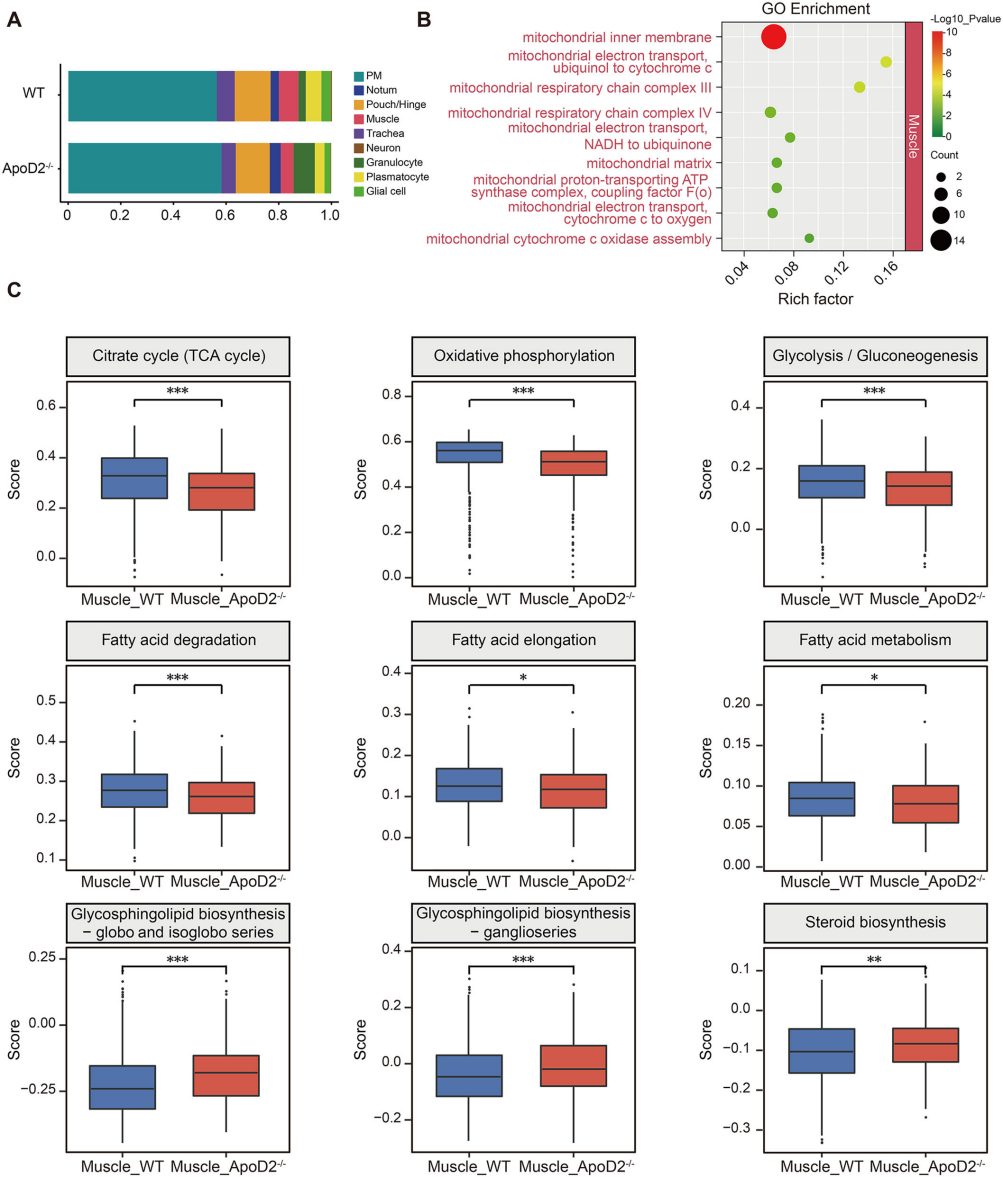
Functional gene enrichment analysis in muscle cell types of wild-type and *ApoD2*^−/−^ mutants. (**A**) Proportional representation of each cell type in the silkworm wing. (**B**) GO functional analysis of DEGs in muscle cell types. (**C**) Gene set enrichment analysis (ssGSEA) of metabolic pathways in muscle cells from WT and *ApoD2*^−/−^ wings. Box plots show the median (center line, 50th percentile), 25th percentile (Q1, bottom edge of box) and 75th percentile (Q3, top edge of box); whiskers extend to the minimum and maximum values. These analyses were performed at the single-cell level, with statistical comparisons conducted across individual cells (*n* = 5,625 cells for WT, *n* = 5181 cells for *ApoD2*^−/−^). Statistical significance was determined by two-sided Wilcoxon rank-sum test. Exact *p* values: citrate cycle (TCA cycle), *p* = 5.00e-8; oxidative phosphorylation, *p* = 1.85e-13; glycolysis/gluconeogenesis, *p* = 6.20e-4; fatty acid degradation, *p* = 6.77e-4; fatty acid elongation, *p* = 1.09e-2; fatty acid metabolism, *p* = 1.38e-2; glycosphingolipid biosynthesis (globo and isoglobo series), *p* = 8.24e-9; glycosphingolipid biosynthesis (ganglio series), *p* =  4.50e-4; steroid biosynthesis, *p* = 7.30e-3. *p < 0.05, **p < 0.01, ***p < 0.001. Source data are available online for this figure.

**Figure EV6:**
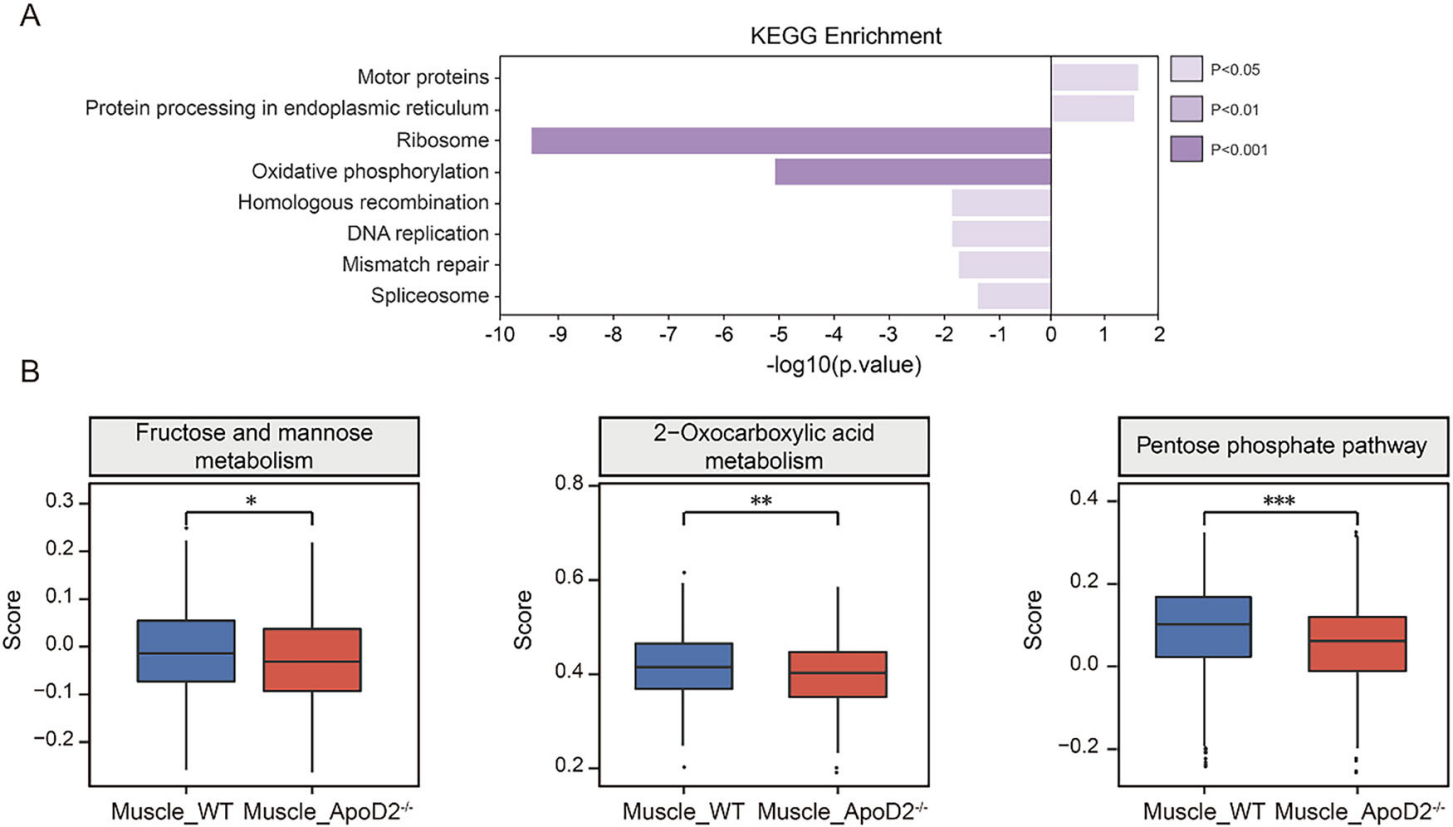
Pathway analysis in muscle cell types of wildtype and *ApoD2*^*−/−*^ mutant. (**A**) KEGG pathway analysis of DEGs in muscle cell types. (**B**) Supplementary single-sample gene set enrichment analysis (ssGSEA) in the muscle cell type of wild-type and *ApoD2*^*−/−*^ mutants.

**Figure EV7:**
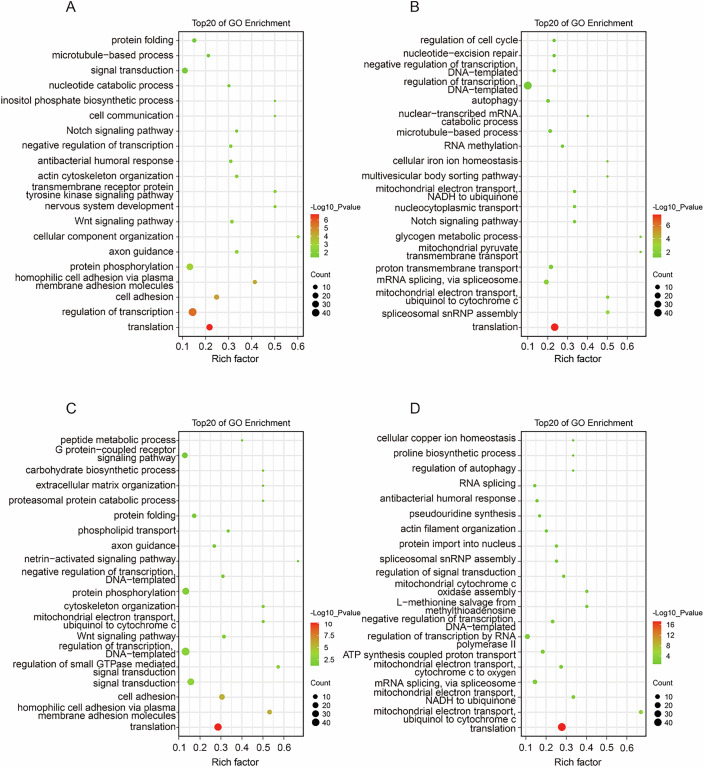
Top 20 results of GO enrichment analysis of differentially expressed genes in four wing cell types of silkworm. Vertical coordinate biological function of the gene or the pathway in which each molecule exerts its function, horizontal coordinate the percentage of the number of genes enriched to the target pathway, the size of the point indicates the number of genes enriched to the gene in each entry, and the significance of the point’s enrichment.

While overall cell-type composition was largely conserved, *ApoD2* mutants exhibited a marked depletion of muscle cells accompanied by an expansion of glial populations (Fig. 4A). Transcriptomic signatures of mutant muscle cells revealed extensive downregulation of genes involved in oxidative phosphorylation, the electron transport chain and autophagy, suggesting potential alterations in mitochondrial activity (Figs. 4B and EV6). Quantitative analysis revealed a marked increase in the number of DEGs associated with autophagy and energy metabolic processes in all three wing cell subtypes (peripodial membrane, notum, and pouch/hinge cells) following *ApoD2* knockout (Fig. EV7; Datasets EV5–9). Gene set enrichment analysis further demonstrated global repression of energy-yielding pathways—including the tricarboxylic acid cycle, glycolysis, and fatty acid catabolism—coupled with upregulation of lipid anabolic processes, such as glycosphingolipid and steroid biosynthesis (Fig. 4C). These transcriptional changes suggest that ApoD2 loss impair mitochondrial energy metabolism and shift gene expression programs toward lipid biosynthesis, implicating ApoD2 in coordinating lipid utilization with energy gene programs during wing tissue differentiation (Datasets EV10 and 11). Together, these findings indicate that ApoD2 is indispensable for maintaining metabolic homeostasis in developing flight muscles.

### ApoD2 coordinates lipid utilization and energy metabolism in wing epithelial cells

**Figure EV8:**
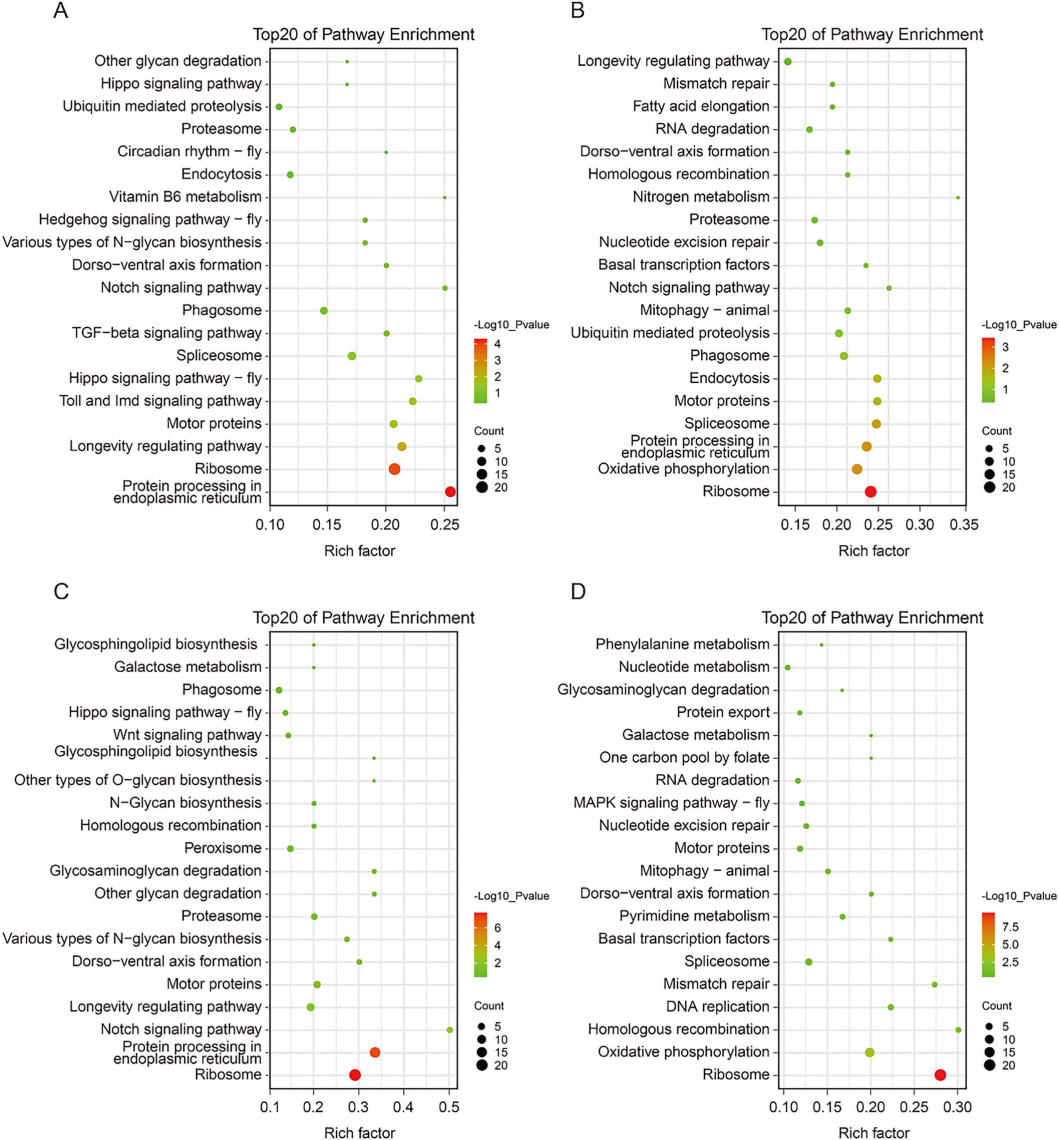
Top 20 results of KEGG enrichment analysis of differentially expressed genes in four wing cell types of silkworm. Vertical coordinate biological function of the gene or the pathway in which each molecule exerts its function, horizontal coordinate the percentage of the number of genes enriched to the target pathway, the size of the point indicates the number of genes enriched to the gene in each entry, and the significance of the point’s enrichment.

**Figure EV9:**
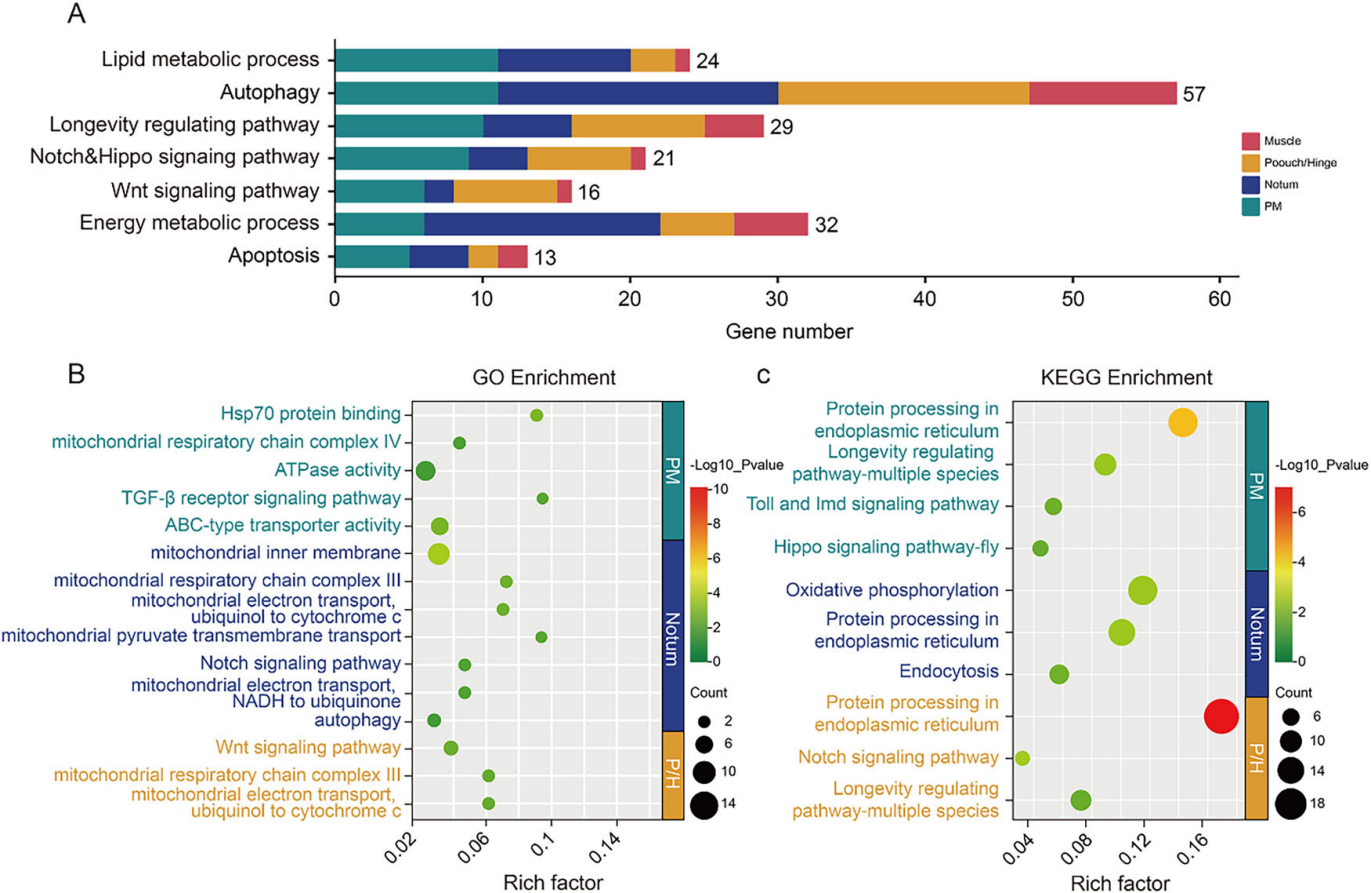
Statistical analysis of differentially expressed genes in three wing cell types of the silkworm. (**A**) Bar plot showing the number of differentially expressed genes (DEGs) associated with specific pathways. The x-axis represents the number of genes, and the y-axis lists the pathways: Lipid metabolic process (lipid metabolism-related genes), Autophagy (autophagy regulation-related genes), Longevity regulating pathway (lifespan regulation-related genes), Notch & Hippo signaling pathway (Notch and Hippo pathway-related genes), Wnt signaling pathway (Wnt pathway-related genes), Energy metabolic process (energy metabolism-related genes), and Apoptosis (apoptosis regulation-related genes). (**B**, **C**) Partial GO and KEGG enrichment analysis of DEGs in three wing cell types of silkworm. The top 20 enriched terms related to lipid metabolism, autophagy, energy metabolism, wing development, and lifespan regulation are displayed. The y-axis represents the biological functions or pathways, and the x-axis represents the percentage of genes enriched in each pathway. Dot size indicates the number of genes enriched in each term, and color intensity reflects the significance of enrichment.

**Figure EV10:**
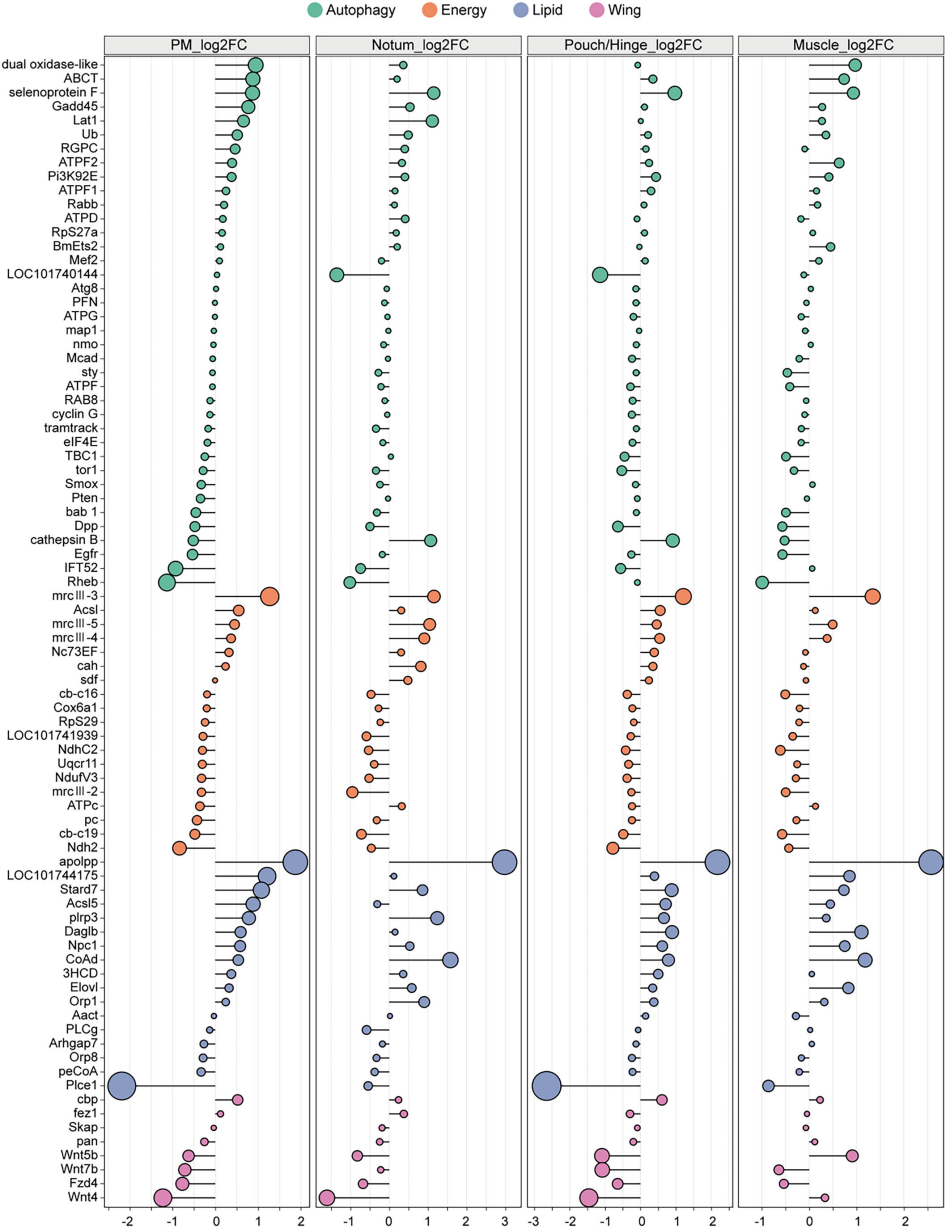
Comparison of lipid, energy, autophagy and wing development-related differential gene expression in four wing cell types of silkworm. Vertical coordinates are the different differential genes, and horizontal coordinates are Log_2_FC values; Log_2_FC >0, upregulates gene expression; Log_2_FC <0, downregulates gene expression.

To dissect the cellular programs governed by ApoD2 during wing development, we examined transcriptional alterations across three key epithelial subtypes—the peripodial membrane (PM), pouch/hinge, and notum cells. Across these cell types, ApoD2 knockout triggered a broad transcriptional reorganization dominated by genes involved in lipid metabolism, autophagy, and energy homeostasis, as well as major developmental signaling cascades including Notch, Hippo, and Wnt (Figs. EV7–10; Datasets EV9–12).

Strikingly, genes encoding components of the mitochondrial electron transport chain and tricarboxylic acid (TCA) cycle were consistently downregulated in all epithelial domains, pointing to transcriptional downregulation of mitochondrial metabolic programs, consistent with reduced mitochondrial activity. In contrast, lipid anabolic and lifespan-associated genes were upregulated, suggesting a compensatory shift from energy production to lipid storage and stress tolerance. The enrichment of autophagy-related transcripts, particularly in the notum and pouch/hinge regions, further indicates an adaptive metabolic response to energetic stress.

Notch signaling was preferentially activated in the notum and pouch/hinge, while TGF-β and Toll/Imd pathways were specifically enriched in PM cells. These patterns imply that ApoD2 loss perturbs the metabolic underpinnings of epithelial differentiation and immune modulation. Together, these results reveal that ApoD2 plays a central role in regulating lipid and energy gene programs during wing epithelial morphogenesis. Its absence is associated with transcriptional suppression of mitochondrial metabolic pathways, activation of autophagy-related gene programs, and a shift in gene expression toward lipid biosynthesis—collectively suggesting impairment of the cellular programs required for proper wing formation.

### Loss of ApoD2 remodels cellular composition and transcriptional networks in developing wings

**Figure 5:**
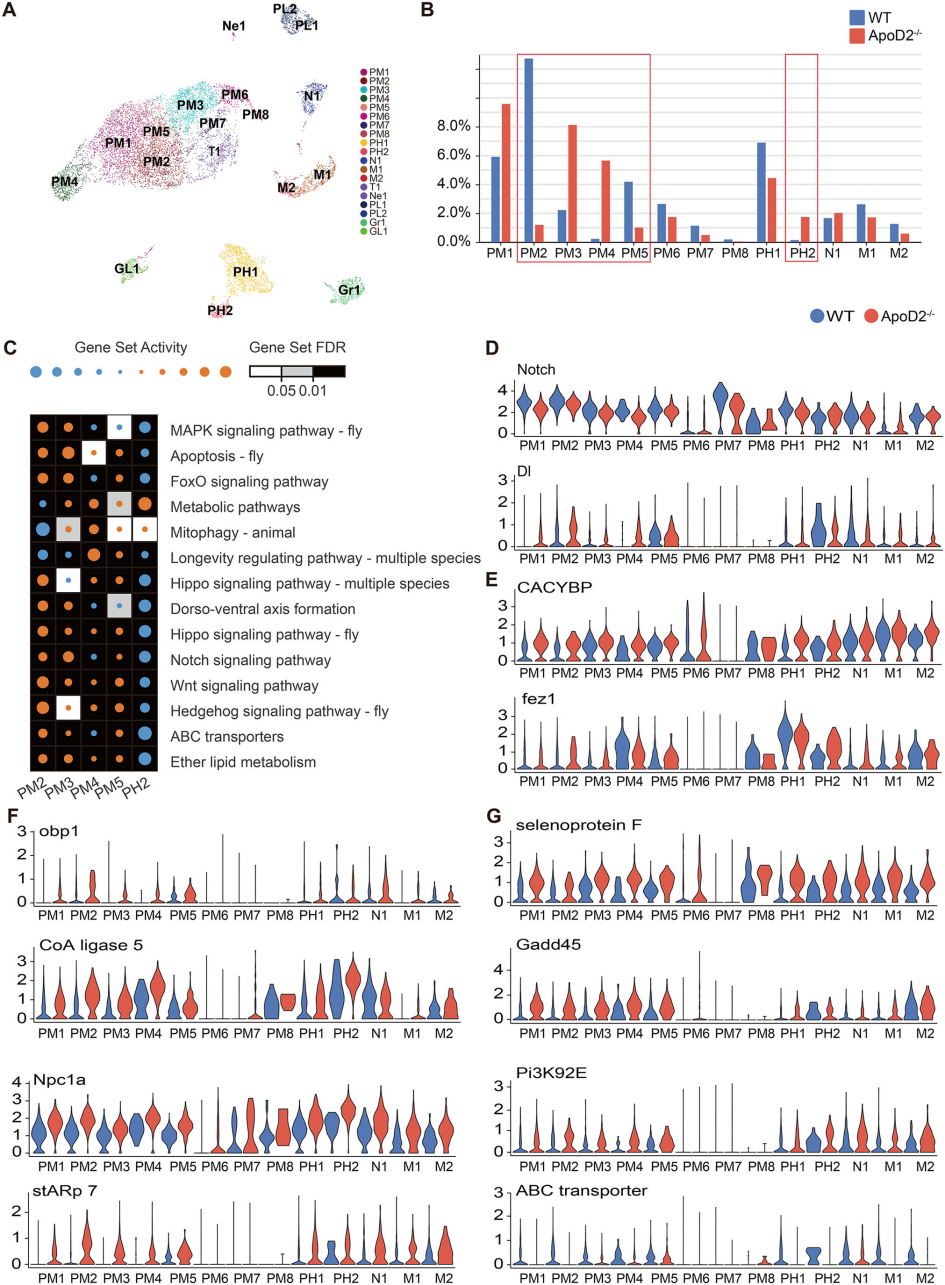
scRNA-seq analysis of wild-type and *ApoD2*^−/−^ mutant wings at the cell cluster level. (**A**) Uniform manifold approximation and projection (UMAP) clustering identified 18 overlapping clusters. (**B**) Proportion of cells in each cluster for WT (blue) and *ApoD2*^−/−^ mutant (red) across the four wing cell types. (**C**) Pathway enrichment analysis of clusters PM2–PM5 and PH2. (**D**) Violin plots showing cluster-specific changes in gene expression for the Notch signaling pathway. (**E**) Violin plots depicting cluster-specific changes in gene expression for cell development-related genes. (**F**) Violin plots illustrating cluster-specific changes in gene expression for lipid-related pathways. (**G**) Violin plots displaying cluster-specific changes in gene expression for autophagy-related pathways. Data information: In (**D**–**G**), analyses were performed at the single-cell level. The number of cells per cluster (*ApoD2*^−/−^, WT): PH1 (480, 746), PH2 (189, 16), PM1 (1,036, 640), PM2 (130, 1,376), PM3 (879, 241), PM4 (612, 25), PM5 (110, 453), PM6 (189, 287), PM7 (54, 123), PM8 (6, 21), M1 (186, 284), M2 (64, 137), N1 (219, 181). Source data are available online for this figure.

Single-cell transcriptomic profiling identified several distinct cellular clusters within WT and *ApoD2*^−/−^ wing tissues, encompassing four major cell types (epithelial, muscle, tracheal, and glial) (Fig. EV5). Several epithelial subclusters exhibited marked compositional shifts between WT and mutant transcriptomes (Fig. 5A,B). In the PM, PM3 and PM4 cells expanded twofold, whereas PM2 and PM5 populations were reduced by half. Similarly, pouch/hinge (PH2) cells dominated the corresponding mutant cluster, while the notum and muscle populations remained stable. These results suggest that ApoD2 loss selectively perturbs epithelial cell fate specification during wing morphogenesis.

To uncover the molecular basis of these changes, we compared the transcriptional landscapes of affected clusters (PM2–PM5 and PH2). Pathway enrichment revealed broad reprogramming of developmental and metabolic signaling networks, including Hippo, Notch, Wnt, Hedgehog, and ABC transporter pathways—all central to epithelial growth and morphogenesis (Fig. 5C). Notably, Notch signaling displayed divergent regulation. In PM clusters, *Notch* was upregulated while its ligand delta was repressed, whereas both were coordinately induced in PH2, indicating cluster-specific rewiring of intercellular signaling (Fig. 5D).

Beyond developmental pathways, genes controlling autophagy and lipid metabolism were strongly upregulated (Fig. 5E–G; Datasets EV9, 11). Together, these transcriptional shifts point to a metabolic stress response triggered by ApoD2 deficiency, coupling altered lipid handling with compensatory activation of catabolic programs. Collectively, these findings reveal that ApoD2 safeguards epithelial homeostasis by coordinating cell-type balance, metabolic state, and morphogen signaling. Its loss disrupts both the cellular architecture and transcriptional coherence of developing wings.

### ApoD2 is essential for maintaining wing lipid reservoirs

**Figure 6:**
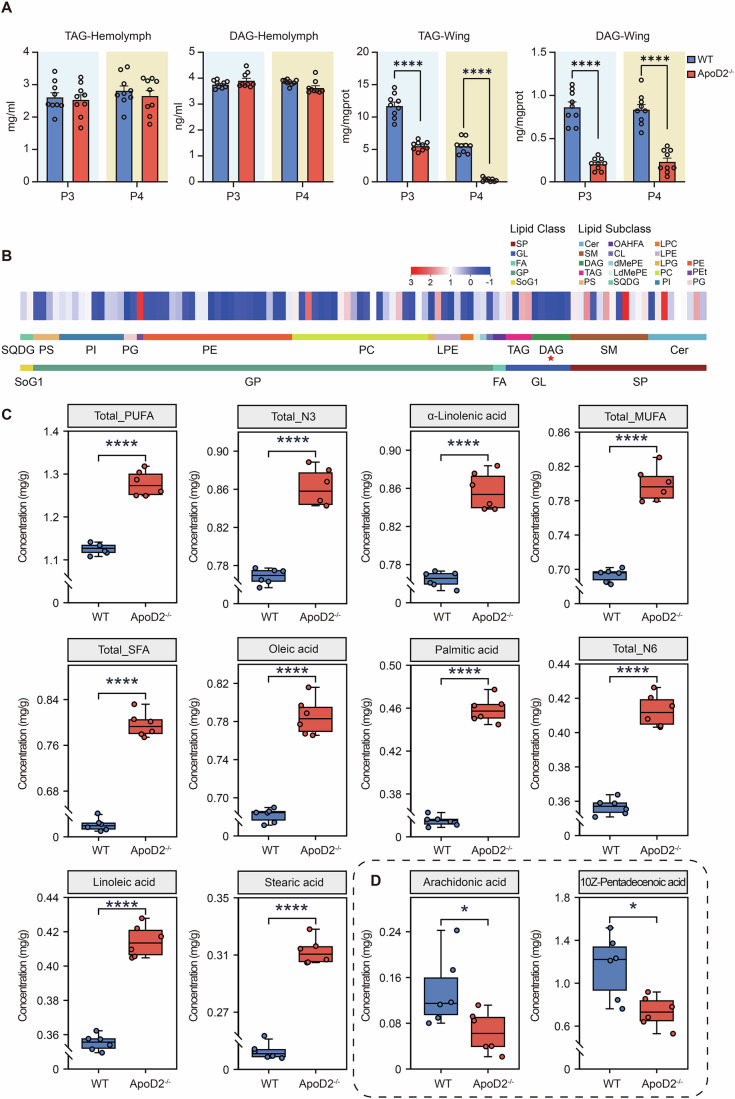
Lipid and fatty acid profiling in wild-type and *ApoD2*^−/−^ mutants. (**A**) Quantification of triacylglycerol (TAG) and diacylglycerol (DAG) levels in the hemolymph and wings of WT and *ApoD2*^−/−^ mutants at P3 and P4 stages. Data represent the mean ± SEM from three biological replicates (*n* = 3), each measured in triplicate. (**B**) Heatmap of lipid profiles in the wings of WT and *ApoD2*^−/−^ mutants at P3 stage. Red and blue indicate increases and decreases in lipid content, respectively. SQDG sulfated isorhamnosyl diacylglycerol, PS phosphatidylserine, PI phosphatidylinositol, PG phosphatidylglycerol, PE phosphatidylethanolamine, PC phosphatidylcholine, LPE lysophosphatidylethanolamine, TAG triacylglycerol, DAG diacylglycerol, SM sphingomyelin, Cer ceramide, SoG1 sphingosine glucose, GP glycerophospholipid, FA fatty acyls, GL glycerol esters, SP sphingolipids. (**C**) Changes in the content of major fatty acids in the hemolymph. Box plots show the median (center line, 50th percentile), 25th percentile (Q1, bottom edge of box) and 75th percentile (Q3, top edge of box); whiskers extend to the minimum and maximum values. total_PUFA total polyunsaturated fatty acids, total_N3 total n-3 polyunsaturated fatty acids, α-Linolenic acid linolenic acid, total_MUFA total monounsaturated fatty acids, total_SFA total saturated fatty acids, oleic acid oleic acid, Palmitic acid, palmitic acid, Total_N6 total n-6 polyunsaturated fatty acids, Linoleic acid linoleic acid; Stearic acid stearic acid. (**D**) Changes in the content of major fatty acids in the wing. Data information: (**A**) statistical significance was determined using two-way ANOVA followed by Tukey’s multiple comparisons test (*n* = 3 biological replicates, each measured in triplicate). Exact adjusted *p* values: TAG-Hemolymph: P3, *p* = 0.9878; P4, *p* =  0.8987. TAG-Wing: P3, *p* < 0.0001; P4, *p* < 0.0001. DAG-Hemolymph: P3, *p* = 0.4662; P4, *p* = 0.1769. DAG-Wing: P3, *p* < 0.0001; P4, *p* < 0.0001. (**C**, **D**) Statistical significance was determined using an unpaired two-tailed Student’s *t*-test (*n* = 6 independent replicates). Exact *p* values for (**C**): total_PUFA, *p* =  3.76e-7; total_N3, *p* = 9.41e-7; α-Linolenic acid, *p* = 9.35e-7; total_MUFA, *p* = 2.33e-7; total_SFA, *p* = 6.90e-9; Oleic acid, *p* = 2.31e-7; Palmitic acid, *p* = 2.20e-9; total_N6, *p* = 7.67e-8; Linoleic acid, *p* = 7.38e-8; Stearic acid, *p* = 1.98e-8. Exact *p* values for (**D**): Arachidonic acid, *p* = 3.48e-2; 10Z-Pentadecenoic acid, *p* = 1.05e-2. Full *p* values for all 45 fatty acids are provided in DatasetS EV13 and EV14. *p < 0.05, **p < 0.01, ***p < 0.001, ****p < 0.0001; ns not significant. Source data are available online for this figure.

To assess the impact of ApoD2 loss on lipid metabolism, we quantified triglyceride (TAG) and diglyceride (DAG) levels in wings and hemolymph at the P3 and P4 developmental stages. While systemic lipid levels in the hemolymph remained largely unchanged, both TAG and DAG were markedly reduced in *ApoD2*^−/−^ wings (Fig. 6A), indicating a tissue-specific lipid deficiency. Comprehensive lipidomic profiling identified 106 lipid species, with the majority—including key glycerophospholipids (PE, PC, and PI) and glycerolipids (DAGs)—significantly downregulated in mutant wings (Fig. 6B). These results demonstrate that ApoD2 deletion leads to a broad depletion of structural and storage lipids within the wing epithelium, revealing its essential role in sustaining localized lipid abundance.

### ApoD2 mediates fatty acid allocation between hemolymph and wing tissue

**Figure EV11:**
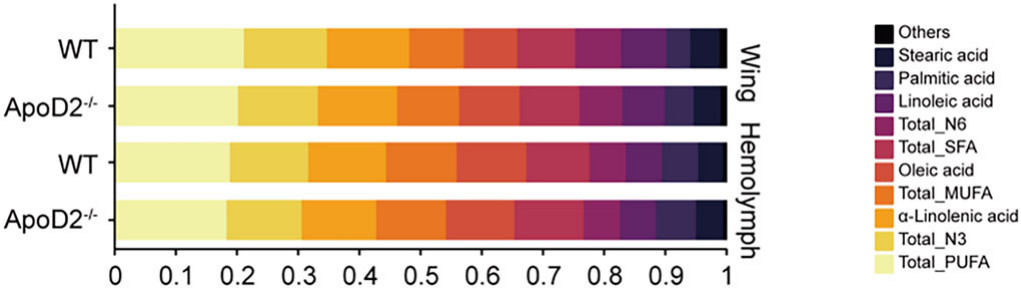
Composition of fatty acids in the wing and hemolymph. Composition of fatty acids in the wing and hemolymph. Stacked bar plots showing the relative proportions of major fatty acid species in the wing and hemolymph of WT and *ApoD2*^−/−^ mutants. Each color represents a different fatty acid class as indicated in the legend.

Targeted metabolomic analysis of 45 mid- and long-chain fatty acids further revealed a striking imbalance in lipid distribution (Fig. EV11 and Datasets EV13 and 14). While total fatty acid composition was unaltered, the absolute abundance of most fatty acids—particularly polyunsaturated (PUFA), monounsaturated (MUFA), and n-3 fatty acids—was significantly elevated in the hemolymph of *ApoD2*^−/−^ mutants (Fig. 6C). In contrast, key fatty acids such as arachidonic acid and 10Z-pentadecenoic acid were depleted in the wing tissue (Fig. 6D). The absence of ApoD2 leads to elevated fatty acid levels in the hemolymph alongside depletion in wing tissue, suggesting a disruption in inter-tissue lipid distribution. Together, these results indicate that ApoD2 is required for the proper allocation of fatty acids between the hemolymph and developing wing, consistent with a role in facilitating lipid availability for local metabolic demands.

### ApoD2 sustains wing energy metabolism

**Figure 7:**
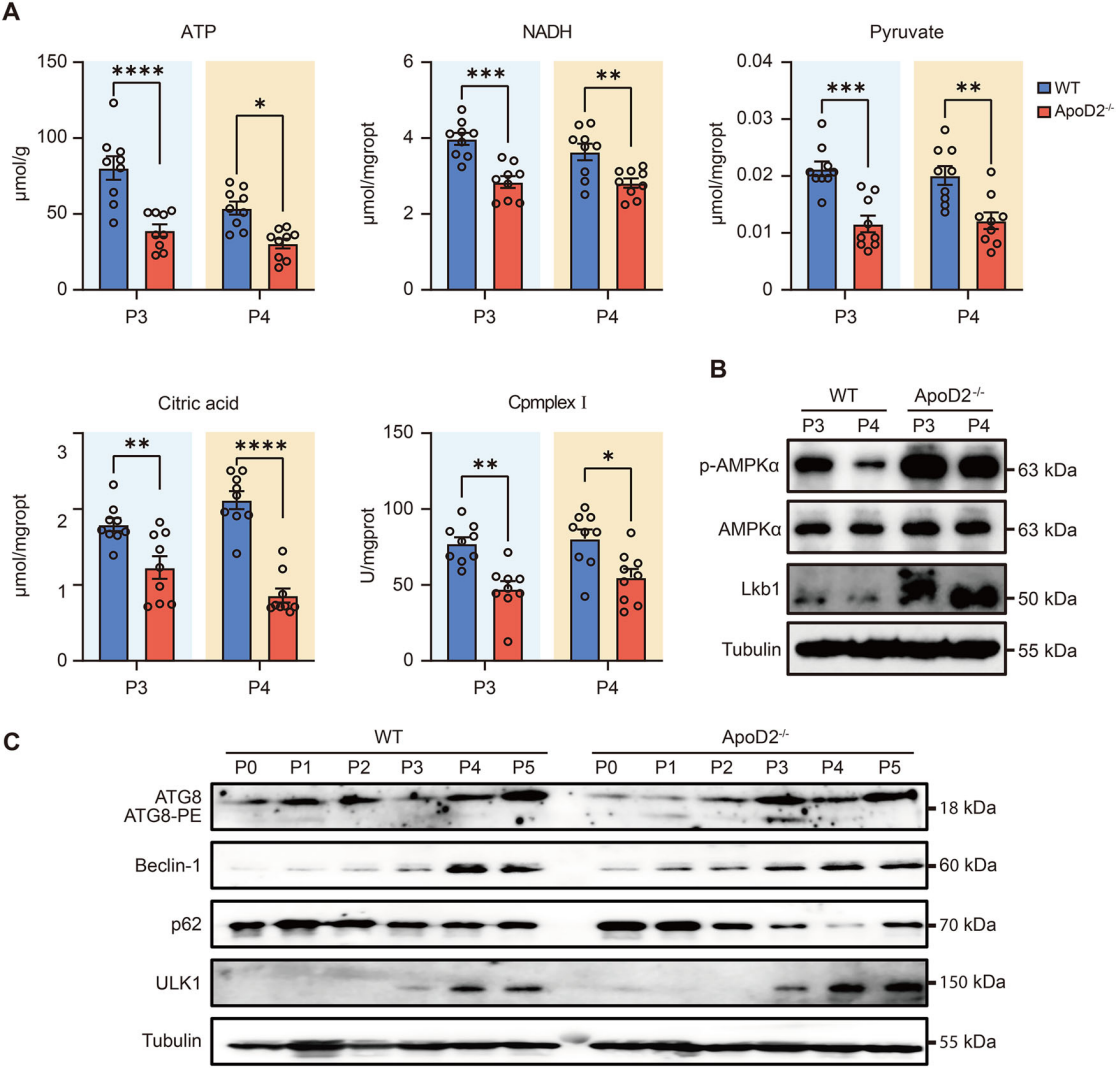
Detection of energy metabolites and autophagy in the wings of wild-type and *ApoD2*^−/−^ mutants. (**A**) Quantification of key energy metabolites—pyruvate, citric acid, NADH, NADH dehydrogenase (Complex I), and ATP—in wings of WT and *ApoD2*^−/−^ mutants at P3 and P4 stages. Data represent the mean ± SEM from three biological replicates (*n* = 3), each measured in triplicate. (**B**) Western blot analysis of AMPK pathway proteins. (**C**) Western blot analysis of autophagy-related proteins. Data information: (**A**), statistical significance was determined using two-way ANOVA followed by Tukey’s multiple comparisons test (*n* = 3 biological replicates, each measured in triplicate). Exact adjusted *p* values: Complex I: P3, *p* = 0.0024; P4, *p* = 0.0118. ATP: P3, *p* < 0.0001; P4, *p* =  0.0146. NADH: P3, *p* = 0.0002; P4, *p* = 0.0078. Citric acid: P3, *p* = 0.0073; P4, *p* < 0.0001. Pyruvate: P3, *p* = 0.0003; P4, *p* =  0.0033. *p < 0.05, **p < 0.01, ***p < 0.001, ****p < 0.0001. Source data are available online for this figure.

Lipids serve as the principal energy substrates during pupal wing development (Nakamura et al, 2014). Consistent with impaired lipid delivery, *ApoD2* knockout caused a marked reduction in key energy metabolites—including pyruvate, citric acid, NADH, NADH dehydrogenase (complex I), and ATP—across both the P3 and P4 stages (Fig. 7A). The decline in NADH and citric acid levels is consistent with reduced activity of β-oxidation and the TCA cycle, while decreased pyruvate and ATP suggest diminished glycolytic and oxidative phosphorylation capacity.

AMP-activated protein kinase (AMPK), a central regulator of cellular energy homeostasis, senses energy status and responds to energy deficits (Mihaylova and Shaw, 2011). These energy deficits caused the activation of the energy-sensing AMPK pathway: both phosphorylated AMPKα and its upstream activator Liver kinase B1 (Lkb1) (Shackelford and Shaw, 2009) were significantly upregulated in *ApoD2*^−/−^ wings (Fig. 7B). Thus, ApoD2 loss is associated with an energy deficit that activates AMPK signaling, likely as a compensatory response to impaired metabolic homeostasis.

### ApoD2 supports wing metabolic homeostasis

Because AMPK activation can initiate autophagy under energy stress (Hu et al, 2021), we next examined molecular hallmarks of autophagy in *ApoD2*^−/−^ wings. Expression of ATG8-PE, Beclin-1, and ULK1 was elevated, whereas the autophagy substrate p62 was reduced (Fig. 7C). These findings indicate that ApoD2 loss is associated with a series of metabolic disturbances—disrupted lipid distribution, lipid depletion within wing tissue, and energy deficits—that are accompanied by autophagy activation. Collectively, these results identify ApoD2 as an important metabolic regulator whose loss perturbs inter-tissue lipid allocation and cellular energy homeostasis, with consequent effects on wing development and morphogenesis.

## Discussion

Our comparative genomic analyses across 791 animal species reveal a striking insect-specific expansion of ApoD homologs, with Lepidoptera exhibiting pronounced lineage-specific duplications. Among these, ApoD2 emerged as a wing-biased paralog carrying a distinctive C-terminal domain predicted to modulate lipid-binding properties. Integrative single-cell transcriptomic, lipidomic, metabolomic, and genetic analyses in *B. mori* demonstrate that ApoD2 neofunctionalization enables spatially resolved lipid allocation critical for wing morphogenesis. These findings establish a mechanistic link between lipid compartmentalization and organ patterning, illustrating how gene duplication can rewire metabolic circuits to drive morphological innovation and reconcile organ-specific energetic demands with developmental complexity.

Metabolic plasticity is an essential evolutionary adaptation that allows organisms to efficiently cope with fluctuating environmental conditions, stabilize community dynamics, and enhance their adaptability to environmental change (Ghedini and Marshall, 2023; Zhang et al, 2024). Single-cell resolution revealed that *ApoD2* loss is associated with compartmentalized transcriptional rewiring: muscle cells show downregulation of fatty acid catabolism genes and upregulation of glycosphingolipid/steroid biosynthesis genes—transcriptional signatures consistent with energy stress (Osipova et al, 2023). While developmental pathways such as Wnt are concomitantly perturbed. ApoD2 may act as a developmental regulator for morphogens like Wnt, analogous to lipophorin’s role in Wingless distribution (Panáková et al, 2005). Cluster-resolved single-cell analysis reveals cell type–specific effects: PM2–PM5 clusters show upregulation of Hippo, Notch, and Hedgehog pathways, whereas PH2 clusters display reciprocal downregulation, linking metabolic perturbation with altered cellular composition and impaired wing morphogenesis. Together, these observations suggest that ApoD2 coordinates metabolic and developmental signaling, although the precise mechanisms remain to be elucidated.

Mechanistically, ApoD2 orchestrates lipid and energy homeostasis in developing wings. Its deletion depletes diacylglycerol, selectively reduces arachidonic acid—a polyunsaturated fatty acid critical for membrane fluidity and signaling (Okada et al, 2013; Prabhakar et al, 2022)—and induces ectopic fatty acid accumulation in the hemolymph. Notably, this redistribution phenotype—wherein hemolymph DAG levels remain largely unaffected while wing DAG and TAG are selectively depleted—argues against a systemic defect in lipophorin-dependent lipid transport and instead implicates ApoD2 in tissue-selective lipid allocation. ApoD2 is a secreted lipocalin containing a conserved calyx-shaped hydrophobic pocket capable of binding fatty acids and signaling lipids (Kielkopf et al, 2018; Marques and Gallazzini, 2024; Sanchez and Ganfornina, 2021). By analogy with vertebrate ApoD, which associates with circulating lipoprotein particles, ApoD2 may transiently interact with lipophorin and operate at the interface between hemolymph circulation and imaginal disc uptake. Collectively, these findings suggest that ApoD2 does not function as a bulk lipid carrier, but rather as a selective lipid transfer protein operating at the interface between hemolymph circulation and imaginal disc uptake, where it modulates the acquisition of lipids by the developing wing disc. ApoD exhibits high binding affinity for signaling lipids such as arachidonic acid and progesterone (Kielkopf et al, 2018), suggesting that ApoD2 may facilitate the targeted allocation of signaling lipids to coordinate lipid homeostasis with developmental signaling during wing morphogenesis. Disruption of this lipid delivery pathway further activates the LKB1–AMPK axis and induces compensatory autophagy (Blenis, 2017), indicating cellular adaptation to reduced lipid-derived energy substrates. Together, these findings suggest that ApoD2 contributes to lipid allocation and energy maintenance in developing wings, with its loss disrupting the coordination between lipid availability and local metabolic demands required for morphogenesis.

The wing defects observed in ApoD2-null *B. mori* are reminiscent of those reported in *D. melanogaster* withered (*whd*) mutants, which lack carnitine palmitoyltransferase I (CPTI), the rate-limiting enzyme for mitochondrial fatty acid β-oxidation (Dark et al, 2024; Strub et al, 2008). Previous studies of *whd* mutants primarily reveal cell-autonomous defects in intracellular lipid utilization. ApoD2 functions as a secreted, tissue-selective lipid transfer protein that directs lipid allocation from the hemolymph to the developing wing disc. Together, these two models outline a continuum of lipid-dependent requirements for wing morphogenesis, underscoring that precise coordination of lipid metabolism at both cellular and organismal levels is essential for proper wing development.

Beyond cellular metabolism, ApoD2 exemplifies how paralog neofunctionalization can link systemic nutrient allocation to organ-specific developmental programs. Its roles in inter-tissue lipid transport and the modulation of morphogen gradients such as Hedgehog and Wnt may together establish a spatially and temporally coordinated metabolic context important for wing formation, though the mechanistic links between these processes remain to be defined. This functional integration resolves a fundamental evolutionary challenge: maintaining metabolic plasticity while supporting biomechanical specialization. The emergence of ApoD2 in Lepidoptera demonstrates how gene duplication and divergence can generate regulatory complexity, enabling lineage-specific innovations in organ function.

More broadly, our findings establish a generalizable framework in which metabolic gene family expansion drives organ-specific energy reprogramming, fostering morphological complexity. Our findings support that metabolic rewiring is a recurrent mechanism for coordinating organ-specific function across diverse lineages (Mantica et al, 2024). Indeed, comparative evidence suggests that analogous neofunctionalization events have shaped organ evolution in vertebrates (Carroll, 2000; Wu et al, 2024), indicating a conserved evolutionary logic linking metabolism and development. Collectively, this work provides a mechanistic blueprint for understanding how metabolic innovation fuels organ diversification, and highlights the evolutionary role of paralog specialization.

## Methods

**Table:** Reagents and tools table

Reagent/resource	Reference or source	Identifier or catalog number
**Experimental models**		
*Bombyx mori*	Zhejiang University	N/A
*Spodoptera frugiperda*	Zhejiang University	N/A
**Antibodies**		
Rabbit anti-p-AMPKα (Ser473)	Cell Signaling Technology	Cat# 2535
Rabbit anti-AMPKα	Abcam	Cat# ab80039
Rabbit anti-Lkb1	HuaBio	Cat# HA500143
Rabbit anti-ATG8	Lu et al, 2023	N/A
Rabbit anti-Beclin-1	Proteintech	Cat# 11306-1-AP
Rabbit anti-p62/SQSTM1	ABclonal	Cat# A18679
Rabbit anti-ULK1	ABclonal	Cat# A8529
Rabbit anti-Tubulin	Wang et al, 2009	N/A
HRP Goat Anti-Rabbit IgG	DINGGUO CHANGSHENG	Cat# IH-0011
**Chemicals, enzymes and other reagents**		
RIPA lysis buffer	Verde Bio (Fryd Bio)	Cat# FD009
PMSF	Shanghai Biotech	Cat# A610425
Dichloromethane	Thermo Fisher	Cat# A454-4
Methanol	Thermo Fisher	Cat# A998-4
Isopropanol	Sinopharm Chemical	Analytical grade
Acetonitrile	Thermo Fisher	N/A
n-Hexane	Sinopharm Chemical	Analytical grade
Ammonium formate	Honeywell Fluka	Cat# 17843-250 G
Formic acid	DIMKA	Cat# 50144-50 mL
BSA	Merck	Cat# 9048-46-8
PBS	Biosharp	Cat# BL601A
DMSO	Biosharp	Cat# BS087
Trizol (RNAiso Plus)	Takara	Cat# 9109
Acridine Orange (AO)	Merck	Cat# 65-61-2
Propidium Iodide (PI)	Merck	Cat# P4170
PVDF membrane	Merck Millipore	Cat# IPVH00010
ECL chemiluminescent substrate	Fdbio Science Biotech	Cat# FD8020
**Software**		
DIAMOND v2.0.11	https://github.com/bbuchfink/diamond	N/A
MAFFT v6.861	https://mafft.cbrc.jp/alignment/software/	N/A
TrimAl v1.4.1	https://vicfero.github.io/trimal/	N/A
IQ-TREE v1.5.5	http://www.iqtree.org/	N/A
iTOL v6.4.3	https://itol.embl.de/	N/A
SOAPnuke v1.4.0	https://github.com/BGI-flexlab/SOAPnuke	N/A
fastp	https://github.com/OpenGene/fastp	N/A
CellRanger v7.1.0	https://www.10xgenomics.com/support/software/cell-ranger	N/A
Seurat v4.1.1	https://satijalab.org/seurat/	RRID: SCR_016341
UMAP v0.2.10.0	https://cran.r-project.org/web/packages/umap/index.html	N/A
DESeq2 v3.20	https://bioconductor.org/packages//release/bioc/html/DESeq2.html	RRID: SCR_015687
LipidSearch v4.1	https://www.thermofisher.com/	N/A
MetaX	https://metax.genomics.cn/	N/A
MSD ChemStation	Agilent Technologies	N/A
TBtools v2.119	https://github.com/CJ-Chen/TBtools-II/releases	N/A
Pheatmap v1.0.12	https://github.com/raivokolde/pheatmap	N/A
GO TermFinder v0.82	https://github.com/gitpan/GO-TermFinder	N/A
GraphPad Prism v9.0	https://www.graphpad.com/features	RRID: SCR_002798
**Other**		
MACS® Tissue Storage Solution	Miltenyi Biotec	Cat# 130-100-008
Chromium Single Cell 3’ V3.1 Reagent Kits	10× Genomics	Cat# PN-1000121
Qubit High Sensitivity DNA Assay	Thermo Fisher Scientific	N/A
Triglyceride Content Assay Kit	SOLARBIO	Cat# BC0625
Diglyceride (DAG) Assay Kit	Laier Bio	Cat# LE-H1996
NAD/NADH Assay Kit	Abcam	Cat# ab65348
Pyruvate Colorimetric/Fluorescence Assay Kit	BioVision	Cat# K609-100
Complex I (NADH-CoQ Reductase) Activity Assay Kit	SOLARBIO	Cat# BC0515
Adenosine Triphosphate (ATP) Assay Kit	SOLARBIO	Cat# BC0300
Citric Acid Content Assay Kit	SOLARBIO	Cat# BC2150
BCA Protein Assay Kit	Fryd Bio	Cat# FD2001

### Multispecies protein database establishment

Genome sequences and annotation files for 11,940 metazoan species, representing 54 taxonomically diverse orders, were obtained from the NCBI database. To ensure comprehensive representation, the longest transcripts were retained by filtering out variable splice variants. A protein database encompassing 11,940 animal species across 54 orders was subsequently constructed using DIAMOND v2.0.11 software.

### Identification of *ApoD* genes in multiple species

*ApoD* genes were identified using *Drosophila ApoD* genes (*Glaz* and *Nlaz*) and the human *ApoD* gene as query sequences. A BLASTP search was performed against the assembled genome databases using DIAMOND v2.0.11 with the following parameters: *E* value ≤1e-10, minimum sequence similarity of 50%, and minimum length match of 80%. To compare the distribution of *ApoD* gene copy numbers across species, a phylogenetic tree was constructed. The number of *ApoD* genes per species was annotated and visualized using iTOL (https://itol.embl.de/).

### Phylogenetic analysis of insect *ApoD* genes

Full-length amino acid sequences of *ApoD* genes from multispecies datasets, specifically those originating from insects, were extracted and aligned using MAFFT v6.861. The aligned sequences were subsequently trimmed with TrimAl v1.4.1 to remove poorly aligned regions. Maximum likelihood phylogenetic trees were reconstructed using IQ-TREE v1.5.5. Branch support was assessed using 10,000 ultrafast bootstrap replicates. Homologous genes from corresponding species were annotated on the phylogenetic trees, and the resulting trees were visualized and annotated using iTOL (https://itol.embl.de/).

### RNA-seq

*B. mori* larvae were reared to L5D3, the following tissues were taken: Brain (Br); Epidermis (Ep); Nerve (Ne); Fat body of male silkworms (mFb), Fat body of female silkworms (fFb); anterior gut (Ag); midgut (Mg); posterior gut (Pg); anterior silk gland (ASG); middle silk gland (MSG); posterior silk gland (PSG); malpighian tubule (Mt); ovary (Ov); testis (Te); trachea (Tr); wing disc (Wd). *Spodoptera frugiperda* larvae were reared to L5D3, and the following different tissues were taken: Epidermis (Ep); fat body (Fb); head (Hd); midgut (Mg); malpighian tubule (Mt); posterior gut (Pg); silk gland (SG); testis (Te); wing disc (Wd). The tissues were subjected to RNA extraction using Trizol and transcriptome sequencing on a BGISEQ-500 sequencer.

### Preparation of single cell suspensions and sequencing

Wing tissues from WT and *ApoD2*^*−/−*^ mutants at the P3 stage were collected and immediately submerged in 1 mL of MACS® Tissue Storage Solution (Cat#: 130-100-008, Miltenyi Biotec) to preserve sample integrity. The cell suspension was filtered through cell strainers. The cell pellet was washed with PBS containing 0.04% bovine serum albumin (BSA). Cell viability was assessed using AO/PI staining and quantified with a Countstar Fluorescence Cell Analyzer.

### Single-cell sequencing

scRNA-Seq libraries were prepared using the 10X Genomics Chromium Controller Instrument and Chromium Single Cell 3’ V3.1 Reagent Kits (10X Genomics). The final libraries were quantified using the Qubit High Sensitivity DNA Assay (Thermo Fisher Scientific). Sequencing was performed on a DNBSEQ-T7 Sequencer (MGI) with a 150 bp paired-end configuration.

### Single-cell data analysis

scRNA-seq data analysis was conducted by NovelBio Co., Ltd. Raw sequencing reads were processed with fastp to filter adapter sequences and remove low-quality reads. Feature-barcode matrices were obtained by aligning reads to the *B. mori* reference genome (Bmori_2016v1.0, Ensembl 56). Downsampling analysis was performed to normalize sequencing depth across samples based on mapped barcoded reads per cell. Cells containing over 200 expressed genes and exhibiting a mitochondrial UMI rate below 20% were retained for downstream analysis.

Data normalization and regression were performed using the Seurat package (v4.1.1). Principal component analysis (PCA) was conducted and the top ten principal components were used for uniform manifold approximation and projection (UMAP) construction. Marker genes for each cluster were identified using the FindAllMarkers function.

### Detection of diglyceride and triglyceride levels

Wing tissues from WT and *ApoD2*^*−/−*^ mutants at the P3 and P4 stages were dissected and collected, with each sample comprising three biological replicates. The weight of each sample was recorded prior to processing. Tissues were homogenized in RIPA Lysis Buffer (FD009, Verde Bio) supplemented with 1% PMSF (A610425, Shanghai Biotech). The levels of diglycerides (DAG) and triglycerides (TAG) were quantified using the Triglyceride Content Assay Kit (BC0625, SOLARBIO) and the Diglyceride Assay Kit (LE-H1996, Laier Bio), respectively, following the manufacturers’ protocols. Lipid concentrations were normalized to the tissue weight of each sample to determine the lipid content per unit weight.

### Wing lipidomics

Wing tissues (*n* = 6 wild-type, *n* = 4 *ApoD2*^−/−^; ~25 mg per sample) at P3 stage were homogenized in 800 µL dichloromethane/methanol (3:1, v/v) containing deuterium-labeled internal standards (15:0-18:1(d7) PC, 18:1(d7) LPE, and 15:0-18:1(d7) PS) spiked prior to homogenization to control for extraction recovery across major glycerophospholipid classes. After tissue lysis (5 min) and ice-bath sonication (10 min), lipids were extracted overnight at −20 °C. Extracts were centrifuged (25,000×*g*, 4 °C, 15 min), and 600 µL supernatant was dried under vacuum and reconstituted in isopropanol/acetonitrile/water (2:1:1, v/v/v). Chromatographic separation was performed on a Waters 2777 C UPLC system with a CSH C18 column (1.7 µm, 2.1 × 100 mm, 55 °C) at 0.4 mL/min (injection volume 5 µL). Positive-mode mobile phases were A: 60% acetonitrile/water with 10 mM ammonium formate and 0.1% formic acid, and B: 90% isopropanol/10% acetonitrile with 10 mM ammonium formate and 0.1% formic acid; negative mode omitted formic acid. A Q Exactive HF mass spectrometer (Thermo Fisher Scientific) was operated in full-scan/data-dependent MS² mode (scan range 70–1050 m/z; resolution 120,000; AGC 3 × 10⁶; top-3 MS², resolution 30,000, NCE 15/30/45 eV). ESI parameters: spray voltage +3.80/−3.20 kV, capillary temperature 320 °C.All reported lipid species were structurally identified using LipidSearch v4.1 (product ion search, ≤5 ppm, grades A–B retained); no uncharacterized ion features were included. Lipids were classified following LIPID MAPS (Fahy et al, 2005; Fahy et al, 2009) and annotated using nomenclature (Liebisch et al, 2020). Abundances were normalized to the nearest-class internal standard and tissue input weight. After QC-RLSC batch correction, KNN imputation, and PQN normalization in MetaX, 354 structurally identified lipid species across 26 subclasses were retained (QC CV <30%). Differential lipids were defined by OPLS-DA (VIP ≥1), fold change (≥1.2 or ≤0.83), and Student’s *t*-test (*p* < 0.05).

### Targeted metabolomics of wings

Wing tissues (~50 mg) and hemolymph from wild-type and *ApoD2*^−/−^ mutants at the P3 stage were thawed at 4 °C and homogenized in 5 mL cold dichloromethane/methanol (2:1, v/v) with vortexing. After washing with 2 mL ultrapure water, the lower organic phase was collected and dried under nitrogen. Isotope-labeled internal standards (NU-CHEK) were spiked prior to extraction. The dried extract was resuspended in 2 mL n-hexane and subjected to acid-catalyzed transmethylation for 30 min to generate fatty acid methyl esters (FAMEs). Following the addition of 2 mL ultrapure water and vortexing, the upper phase (1 mL) was collected, dried under nitrogen, and reconstituted in n-hexane for injection. A pooled QC sample was prepared from equal aliquots of all samples and injected at regular intervals throughout the run. Chromatographic separation was performed on an Agilent 7890B GC system with a DB-23 capillary column (60 m × 250 µm × 0.15 µm) using helium as the carrier gas at 1.0 mL/min. The temperature gradient was as follows: 80 °C initial, ramped to 180 °C at 20 °C/min, then to 280 °C at 5 °C/min. Detection was performed on an Agilent 5977B MSD in Scan/SIM mode with electron impact ionization (70 eV); inlet, ion source, and transfer line temperatures were 280, 230, and 250 °C, respectively. Fatty acids were quantified by isotope dilution against external calibration curves (*R* > 0.99 for all analytes) using MSD ChemStation software and normalized to tissue input weight. All QC samples showed RSD <30% across detected species, confirming analytical reproducibility.

### Detection of energy metabolite levels

Wing tissues from WT and *ApoD2*^*−/−*^ mutants at the P3 and P4 stages were collected, with each sample comprising three biological replicates. The levels of five key energy metabolites—NAD/NADH, pyruvate, NADH-CoQ reductase, ATP, and citric acid—were quantified using the following kits: NAD/NADH Assay Kit (ab65348, Abcam), Pyruvate Colorimetric/Fluorescence Assay Kit (K609-100, BioVision), Complex I Activity Assay Kit (BC0515, SOLARBIO), Adenosine Triphosphate Assay Kit (BC0300, SOLARBIO), and Citric Acid Content Assay Kit (BC2150, SOLARBIO). Metabolite concentrations were normalized to the protein content of each sample to determine the metabolite levels per unit protein.

### Western blot

The following primary antibodies were diluted in 5% skimmed milk solution (1:4000 dilution): p-AMPKα (#2535, Cell Signaling Technology), AMPKα (ab80039, Abcam), Lkb1 (HA500143, HuaBio), ATG8 (Zhejiang Institute of Science and Technology), Beclin-1 (11306-1-AP, Proteintech), p62 (A18679, ABclonal), ULK1 (A8529, ABclonal), and Tubulin (loading control). Horseradish peroxidase (HRP)-conjugated goat anti-rabbit IgG (IH-0011, DINGGUO CHANGSHENG) was used as the secondary antibody. Protein bands were visualized using a chemiluminescent substrate and imaged on a ChemiScope 3200 chemiluminescent gel imaging system (CLINX).

### Statistics and analysis of data

DEGs were identified using DESeq2 v3.20 with the following criteria: |log_2_FC | >1 and *p* value <0.05. Statistical significance was defined with the following annotations: *p < 0.05, **p < 0.01, ***p < 0.001, and ns (not significant) for *p* > 0.05. All experimental data were derived from three independent replicates and are expressed as mean ± SEM.

## Supplementary information

Peer Review File

Dataset EV1

Dataset EV2

Dataset EV3

Dataset EV4

Dataset EV5

Dataset EV6

Dataset EV7

Dataset EV8

Dataset EV9

Dataset EV10

Dataset EV11

Dataset EV12

Dataset EV13

Dataset EV14

Source data Fig. 1

Source data Fig. 2

Source data Fig. 3

Source data Fig. 4

Source data Fig. 5

Source data Fig. 6

Source data Fig. 7

Expanded View Figures

## Data Availability

All other data are included in the main text and/or supporting information. Lipidomics data were uploaded to the MetaboLights database (MTBLS11285; https://www.ebi.ac.uk/metabolights/reviewerfdc2372c-1e72-4a17-b79a-fb545bf7d767). Targeted metabolomics data were deposited in the CNCB BioProject database (PRJCA060360; https://ngdc.cncb.ac.cn/omix/preview/dkUPFvDn). The source data of this paper are collected in the following database record: biostudies:S-SCDT-10_1038-S44318-026-00821-0.
