# Supplementary material for: Apolipoprotein D neofunctionalization couples lipid allocation to wing evolution
Source: EMBO J. 2026 Jun 3;45(14):4956–87. doi: 10.1038/s44318-026-00821-0 (PMC13373240; doi:10.1038/s44318-026-00821-0)
Supplement: Supplementary file 23 — Expanded View Figures [file 44318_2026_821_MOESM23_ESM.pdf]

Expanded View Figures

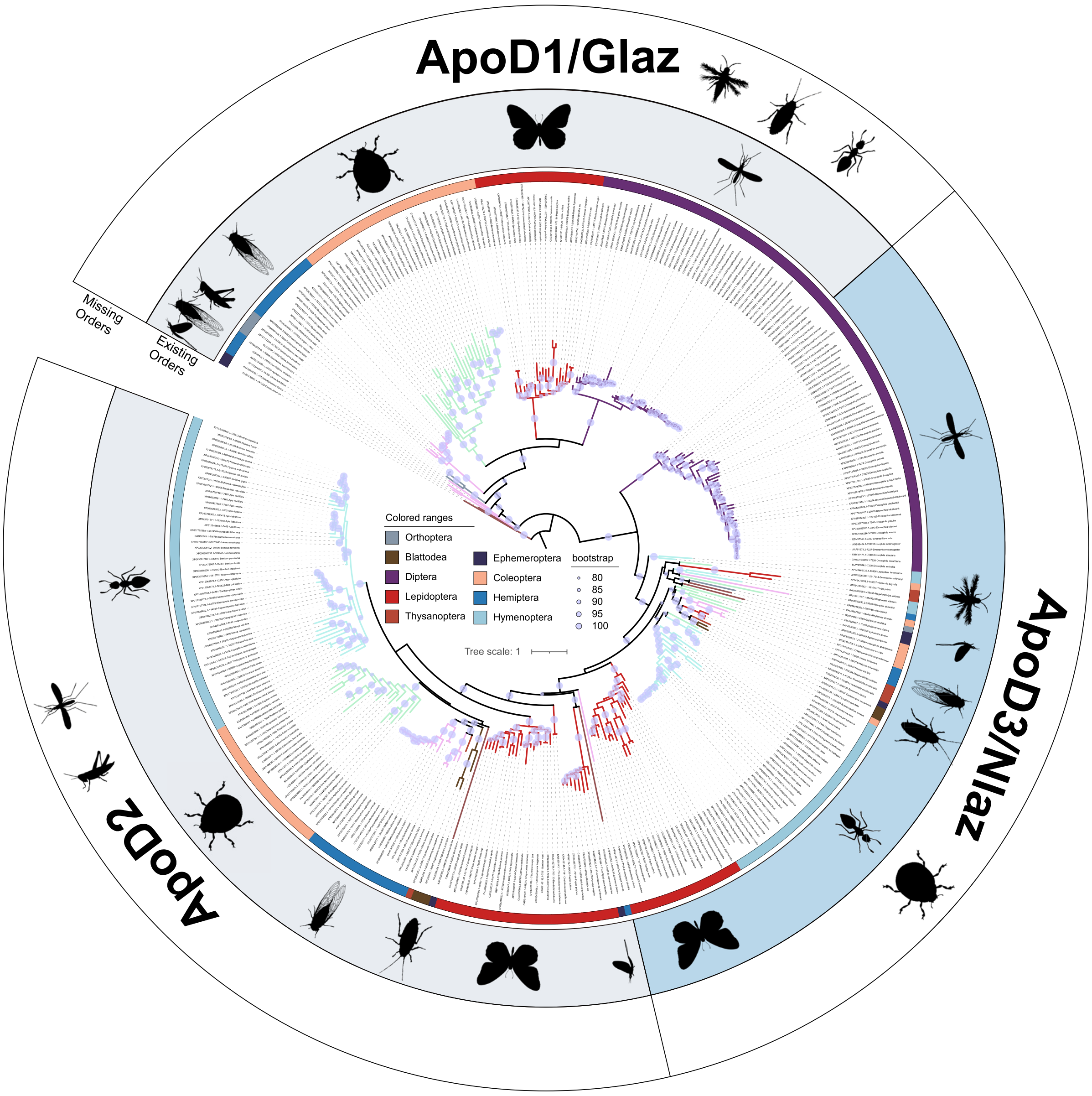

Figure EV1. Phylogenetic tree of *ApoD* genes in the Insecta section.

The colors of the inner circles represent different insect orders, the middle circles represent insect orders with *ApoD* gene subfamilies, and the outermost circles represent different *ApoD* gene subfamilies and insect orders lacking the genes of the subfamily.

 © The Author(s)

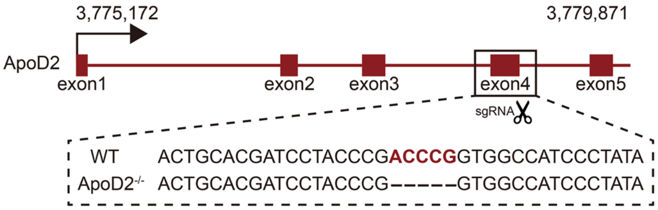

Figure EV2. Schematic diagram of *ApoD2* gene knockout.

The *ApoD2* gene structure showing five exons is illustrated, with the sgRNA target site located in exon 4. The sequence ACCCG is deleted in the *ApoD2*$^{-/-}$ mutant, resulting in a 5-bp deletion.

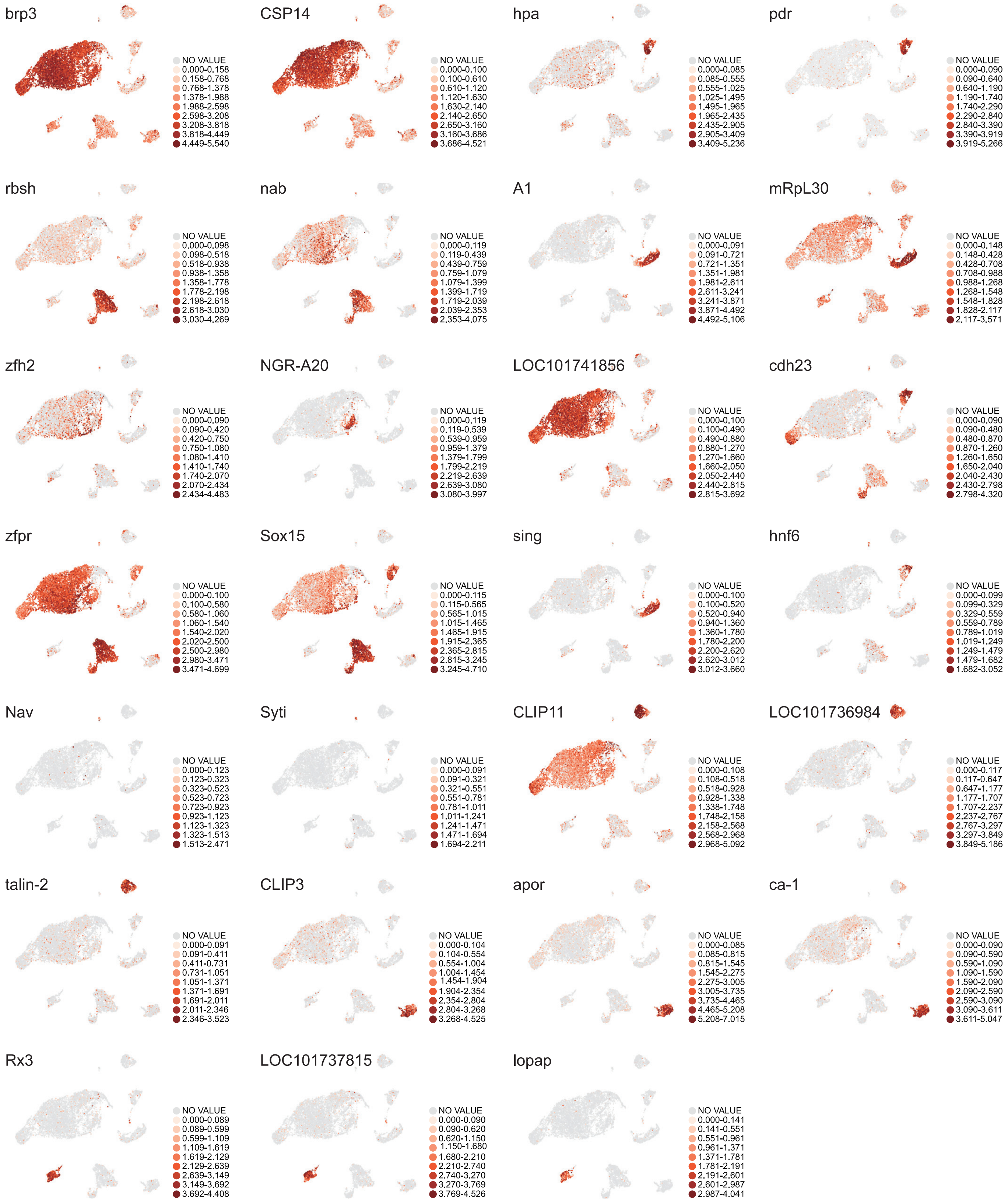
brp3
NO VALUE
0.000-0.158
0.158-0.768
0.768-1.378
1.378-1.988
1.988-2.598
2.598-3.208
3.208-3.818
3.818-4.449
4.449-5.540
CSP14
NO VALUE
0.000-0.100
0.100-0.610
0.610-1.120
1.120-1.630
1.630-2.140
2.140-2.650
2.650-3.160
3.160-3.686
3.686-4.521
hpa
NO VALUE
0.000-0.085
0.085-0.555
0.555-1.025
1.025-1.495
1.495-1.965
1.965-2.435
2.435-2.905
2.905-3.409
3.409-5.236
pdr
NO VALUE
0.000-0.090
0.090-0.640
0.640-1.190
1.190-1.740
1.740-2.290
2.290-2.840
2.840-3.390
3.390-3.919
3.919-5.266
rbsh
NO VALUE
0.000-0.098
0.098-0.518
0.518-0.938
0.938-1.358
1.358-1.778
1.778-2.198
2.198-2.618
2.618-3.030
3.030-4.269
nab
NO VALUE
0.000-0.119
0.119-0.439
0.439-0.759
0.759-1.079
1.079-1.399
1.399-1.719
1.719-2.039
2.039-2.353
2.353-4.075
A1
NO VALUE
0.000-0.091
0.091-0.721
0.721-1.351
1.351-1.981
1.981-2.611
2.611-3.241
3.241-3.871
3.871-4.492
4.492-5.106
mRpL30
NO VALUE
0.000-0.148
0.148-0.428
0.428-0.708
0.708-0.988
0.988-1.268
1.268-1.548
1.548-1.828
1.828-2.117
2.117-3.571
zfh2
NO VALUE
0.000-0.090
0.090-0.420
0.420-0.750
0.750-1.080
1.080-1.410
1.410-1.740
1.740-2.070
2.070-2.434
2.434-4.483
NGR-A20
NO VALUE
0.000-0.119
0.119-0.539
0.539-0.959
0.959-1.379
1.379-1.799
1.799-2.219
2.219-2.639
2.639-3.080
3.080-3.997
LOC101741856
NO VALUE
0.000-0.100
0.100-0.490
0.490-0.880
0.880-1.270
1.270-1.660
1.660-2.050
2.050-2.440
2.440-2.815
2.815-3.692
cdh23
NO VALUE
0.000-0.090
0.090-0.480
0.480-0.870
0.870-1.260
1.260-1.650
1.650-2.040
2.040-2.430
2.430-2.798
2.798-4.320
zfpr
NO VALUE
0.000-0.100
0.100-0.580
0.580-1.060
1.060-1.540
1.540-2.020
2.020-2.500
2.500-2.980
2.980-3.471
3.471-4.699
Sox15
NO VALUE
0.000-0.115
0.115-0.565
0.565-1.015
1.015-1.465
1.465-1.915
1.915-2.365
2.365-2.815
2.815-3.245
3.245-4.710
sing
NO VALUE
0.000-0.100
0.100-0.520
0.520-0.940
0.940-1.360
1.360-1.780
1.780-2.200
2.200-2.620
2.620-3.012
3.012-3.660
hnf6
NO VALUE
0.000-0.099
0.099-0.329
0.329-0.559
0.559-0.789
0.789-1.019
1.019-1.249
1.249-1.479
1.479-1.682
1.682-3.052
Nav
NO VALUE
0.000-0.123
0.123-0.323
0.323-0.523
0.523-0.723
0.723-0.923
0.923-1.123
1.123-1.323
1.323-1.513
1.513-2.471
Syti
NO VALUE
0.000-0.091
0.091-0.321
0.321-0.551
0.551-0.781
0.781-1.011
1.011-1.241
1.241-1.471
1.471-1.694
1.694-2.211
CLIP11
NO VALUE
0.000-0.108
0.108-0.518
0.518-0.928
0.928-1.338
1.338-1.748
1.748-2.158
2.158-2.568
2.568-2.968
2.968-5.092
LOC101736984
NO VALUE
0.000-0.117
0.117-0.647
0.647-1.177
1.177-1.707
1.707-2.237
2.237-2.767
2.767-3.297
3.297-3.849
3.849-5.186
talin-2
NO VALUE
0.000-0.091
0.091-0.411
0.411-0.731
0.731-1.051
1.051-1.371
1.371-1.691
1.691-2.011
2.011-2.346
2.346-3.523
CLIP3
NO VALUE
0.000-0.104
0.104-0.554
0.554-1.004
1.004-1.454
1.454-1.904
1.904-2.354
2.354-2.804
2.804-3.268
3.268-4.525
apor
NO VALUE
0.000-0.085
0.085-0.815
0.815-1.545
1.545-2.275
2.275-3.005
3.005-3.735
3.735-4.465
4.465-5.208
5.208-7.015
ca-1
NO VALUE
0.000-0.090
0.090-0.590
0.590-1.090
1.090-1.590
1.590-2.090
2.090-2.590
2.590-3.090
3.090-3.611
3.611-5.047
Rx3
NO VALUE
0.000-0.089
0.089-0.599
0.599-1.109
1.109-1.619
1.619-2.129
2.129-2.639
2.639-3.149
3.149-3.692
3.692-4.408
LOC101737815
NO VALUE
0.000-0.090
0.090-0.620
0.620-1.150
1.150-1.680
1.680-2.210
2.210-2.740
2.740-3.270
3.270-3.769
3.769-4.526
lopap
NO VALUE
0.000-0.141
0.141-0.551
0.551-0.961
0.961-1.371
1.371-1.781
1.781-2.191
2.191-2.601
2.601-2.987
2.987-4.041

© The Author(s)

Figure EV3. Expression of marker genes in each cell type.

Epithelial cells were identified using the marker gene *pgh* (Deng et al, 2021; Ma et al, 2017), while muscle cells were identified by the expression of *Kettin* (Bullard et al, 2002; Lakey et al, 1993), *A1* (Timson et al, 1998; Wawro et al, 2022), *mRpL30* (Cheong et al, 2020; Rai et al, 2024), and *sing* (Brunetti et al, 2015). Tracheal cells were labeled with *zfh2* (Perea et al, 2013; Terriente et al, 2008) and *NGR-A20* (Grundemar, 1997; Wu et al, 2024), while neurons were labeled with *hnf6* (Audouard et al, 2012; Francius and Clotman, 2010), *Nav* (Feng et al, 1995; Planells-Cases et al, 2000), and *Syti* (Liu et al, 2009; Ro et al, 2010). Glial cells were labeled with *lopap* (Fritzen et al, 2005; Reis et al, 1999) and *Rx3* (Kraft et al, 2016; Zagozewski et al, 2014), and immune-related cell types included plasmatocytes labeled with *CLIP11* (Kanost and Jiang, 2015) and *talin-2* (von Essen et al, 2016) and granulocytes labeled with *CLIP3* (Vaseeharan et al, 2011; Zhang et al, 2018), *apor* (Brankatschk and Eaton, 2010; Canavoso and Rubiolo, 1994), and *ca-1* (Pipoly and Crouch, 1987).

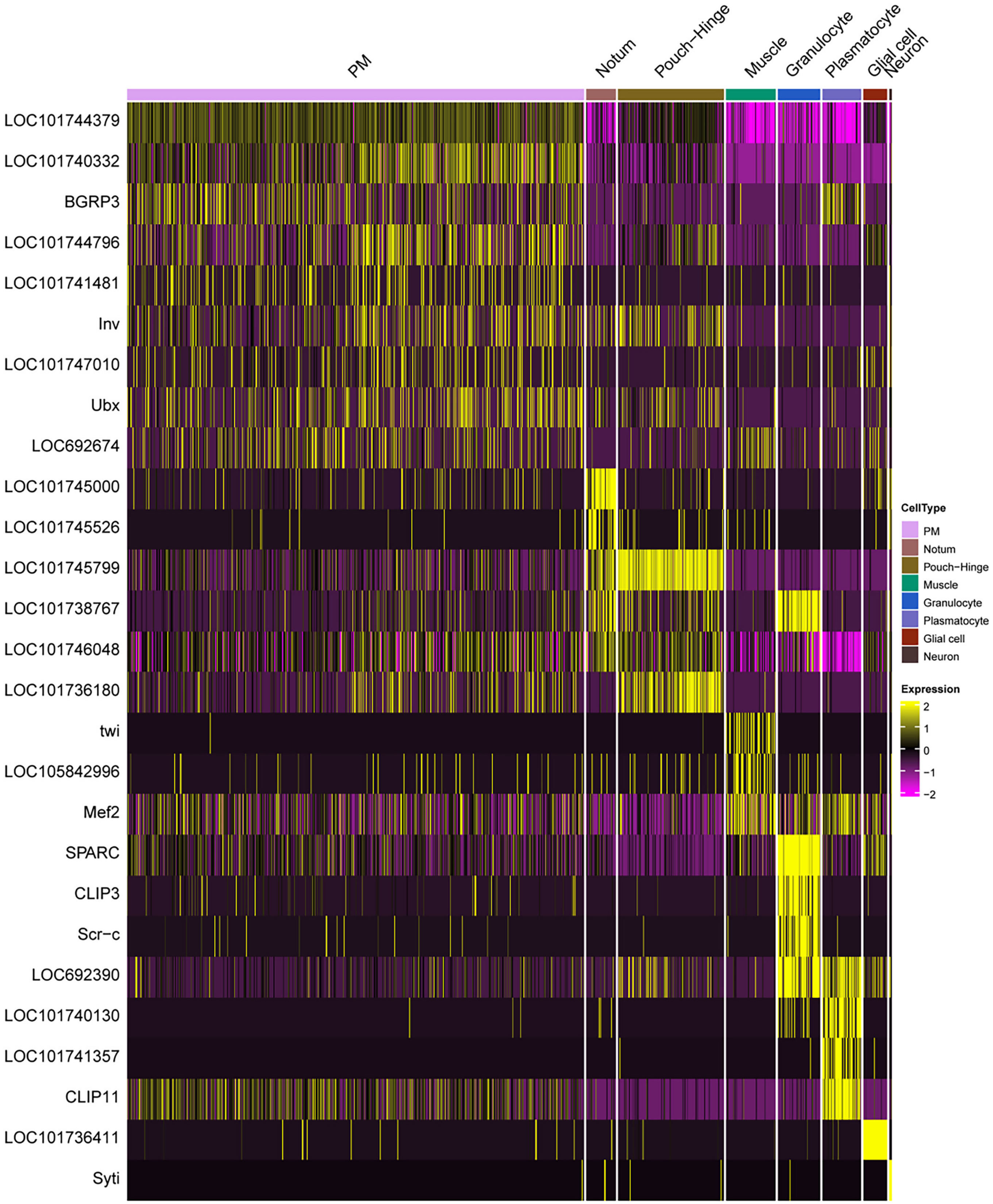
PM
Notum
Pouch−Hinge
Muscle
Granulocyte
Plasmatocyte
Glial cell
Neuron
LOC101744379
LOC101740332
BGRP3
LOC101744796
LOC101741481
Inv
LOC101747010
Ubx
LOC692674
LOC101745000
LOC101745526
LOC101745799
LOC101738767
LOC101746048
LOC101736180
twi
LOC105842996
Mef2
SPARC
CLIP3
Scr−c
LOC692390
LOC101740130
LOC101741357
CLIP11
LOC101736411
Syti
CellType
PM
Notum
Pouch−Hinge
Muscle
Granulocyte
Plasmatocyte
Glial cell
Neuron
Expression
2
1
0
−1
−2

 © The Author(s)

Figure EV4. Heatmap of the proportional expression values of the top ten most upregulated genes in different cell types of silkworm wing tissue.

Heatmap of the proportional expression values of the top upregulated genes in different cell types of silkworm wing tissue. Each column represents a single cell, and each row represents a gene. Colors indicate scaled expression levels, ranging from high (yellow) to low (purple). Cell types are indicated by colored bars at the top of the heatmap.

© The Author(s)

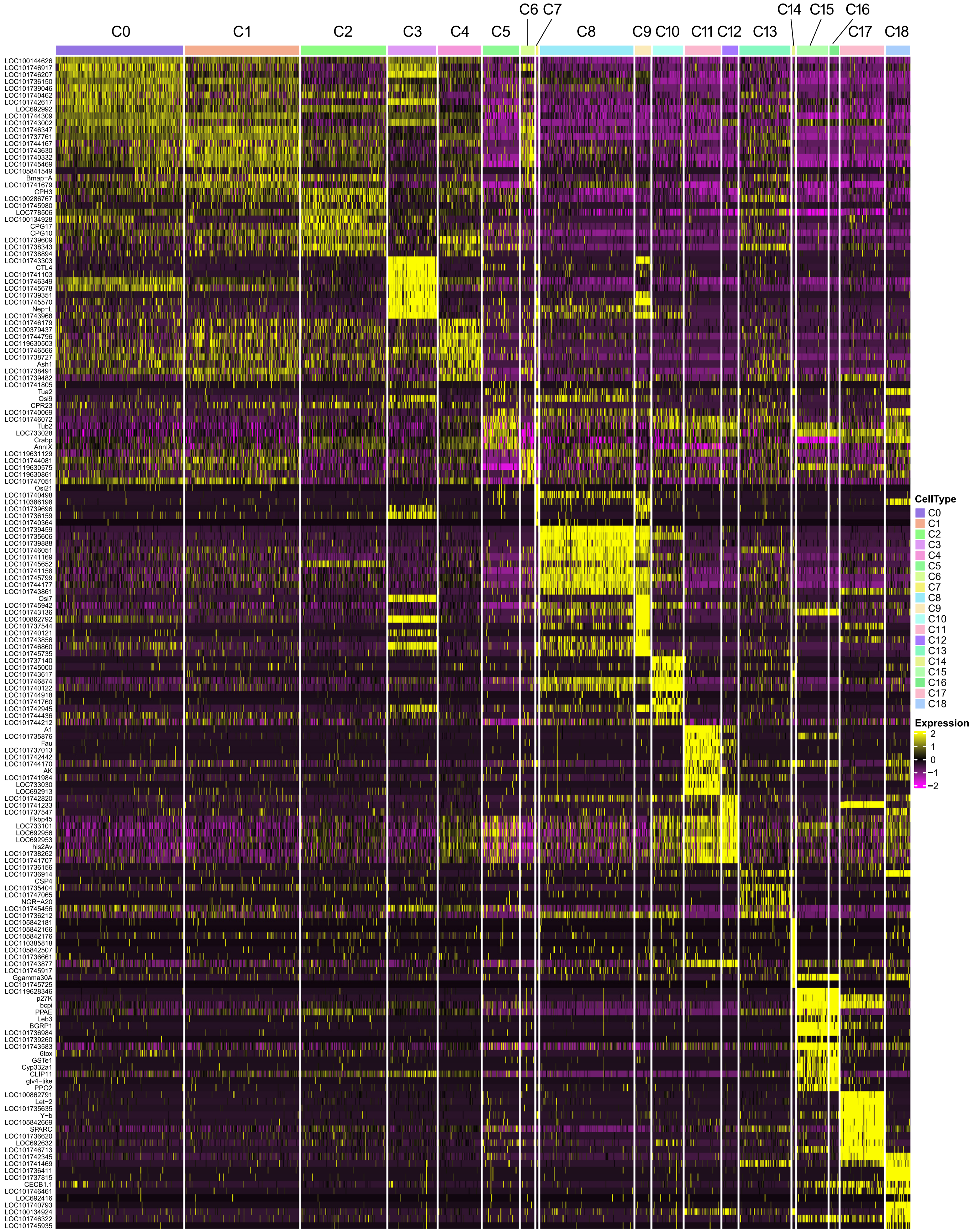
C0
C1
C2
C3
C4
C5
C6 C7
C8
C9 C10
C11 C12
C13
C14 C15 C16
C17
C18
LOC100144626
LOC101746917
LOC101746207
LOC101736150
LOC101739046
LOC101740462
LOC101742617
LOC692992
LOC101744309
LOC101743002
LOC101746347
LOC101737761
LOC101744167
LOC101743630
LOC101740332
LOC101745469
LOC105841549
Bmap−A
LOC101741679
CPH3
LOC100286767
LOC101745980
LOC778506
LOC100134928
CPG17
CPG10
LOC101739609
LOC101738343
LOC101738894
LOC101743303
CTL4
LOC101741103
LOC101746349
LOC101745678
LOC101739351
LOC101745570
Nep−L
LOC101743968
LOC101746179
LOC100379437
LOC101744796
LOC119630503
LOC101746566
LOC101738727
Ash1
LOC101738491
LOC101739482
LOC101741805
Tua2
Osi9
CPR23
LOC101740069
LOC101746072
Tub2
LOC733028
Crabp
AnnIX
LOC119631129
LOC101744081
LOC119630575
LOC119630861
LOC101747051
Osi21
LOC101740498
LOC110386198
LOC101739696
LOC101736159
LOC101740364
LOC101739459
LOC101735606
LOC101739888
LOC101746051
LOC101741169
LOC101745652
LOC101741158
LOC101745799
LOC101744177
LOC101743861
Osi7
LOC101745942
LOC101743136
LOC100862792
LOC101737544
LOC101740121
LOC101743856
LOC101746860
LOC101745735
LOC101737140
LOC101745000
LOC101743617
LOC101746874
LOC101740122
LOC101744918
LOC101741760
LOC101742945
LOC101744436
LOC101744212
A1
LOC101735876
Fau
LOC101737013
LOC101742442
LOC101744170
AK
LOC101741984
LOC733030
LOC692913
LOC101742820
LOC101741233
LOC101737547
Fkbp45
LOC733101
LOC692956
LOC692953
his2Av
LOC101738262
LOC101741707
LOC101736156
LOC101736914
CSP4
LOC101735404
LOC101747065
NGR−A20
LOC101745456
LOC101736212
LOC105842181
LOC105842166
LOC105842176
LOC110385818
LOC105842507
LOC101736661
LOC101743877
LOC101745917
Ggamma30A
LOC101745725
LOC119628346
p27K
bcpi
PPAE
Leb3
BGRP1
LOC101736984
LOC101739260
LOC101743583
6tox
GSTe1
Cyp332a1
CLIP11
glv4−like
PPO2
LOC100862791
Let−2
LOC101735635
Y−b
LOC105842669
SPARC
LOC101736620
LOC692632
LOC101746713
LOC101742345
LOC101741469
LOC101736411
LOC101737815
CECB1.1
LOC101746461
LOC692416
LOC101740793
LOC100134924
LOC101746322
LOC101745935
CellType
C0
C1
C2
C3
C4
C5
C6
C7
C8
C9
C10
C11
C12
C13
C14
C15
C16
C17
C18
Expression
2
1
0
−1
−2

 © The Author(s)

Figure EV5. Heatmap of the proportional expression values of the top ten most upregulated genes.

Subclassification of epithelial cells was achieved using markers: *brp3* and *CSP14* for peripodial membrane (PM); *hpa* (Gómez-Skarmeta and Modolell, 1996), *pdr*, and *cdh23* for notum; *nab*, *zfpr* (Staehling-Hampton et al, 1995; Wu et al, 2019), *rbsh* (Klein et al, 2000), *Sox15* (Song et al, 2019) for pouch/hinge.

© The Author(s)

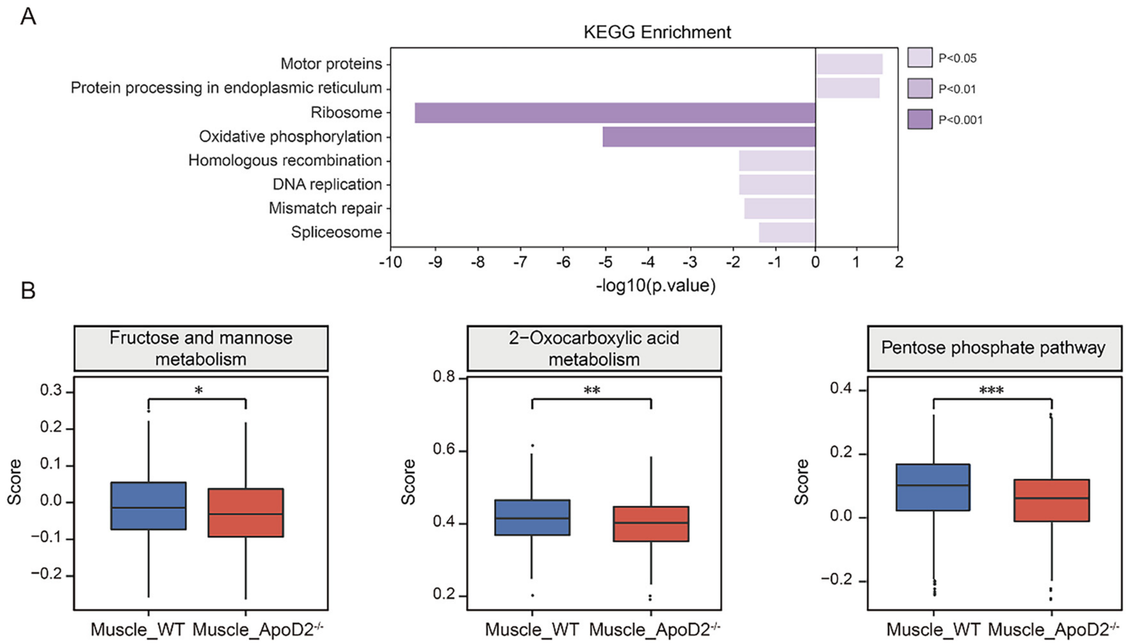

Figure EV6. Pathway analysis in muscle cell types of wildtype and *ApoD2*$^{-/-}$ mutant.

(**A**) KEGG pathway analysis of DEGs in muscle cell types. (**B**) Supplementary single-sample gene set enrichment analysis (ssGSEA) in the muscle cell type of wild-type and *ApoD2*$^{-/-}$ mutants.

 © The Author(s)

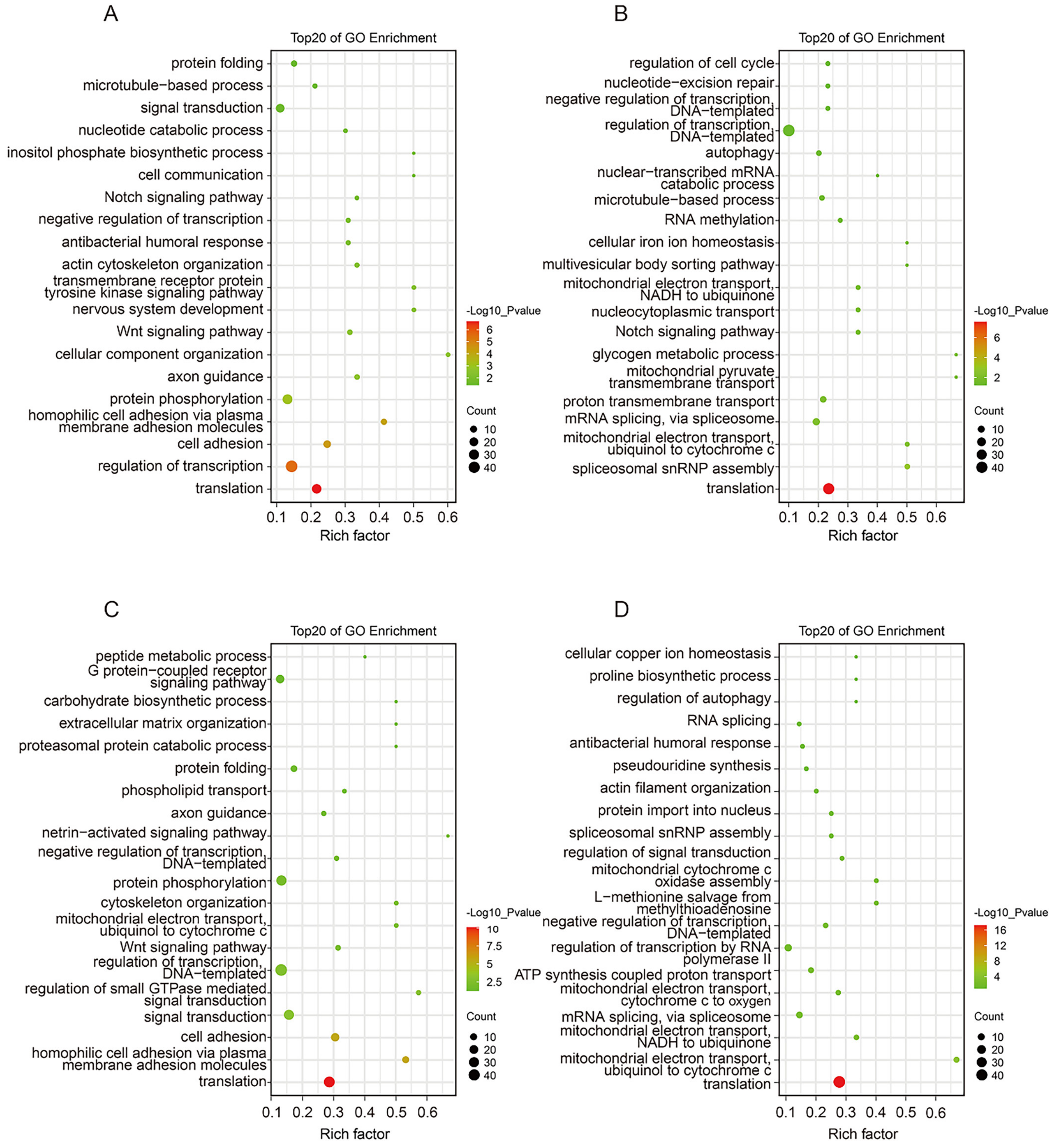

Figure EV7. Top 20 results of GO enrichment analysis of differentially expressed genes in four wing cell types of silkworm.

Vertical coordinate biological function of the gene or the pathway in which each molecule exerts its function, horizontal coordinate the percentage of the number of genes enriched to the target pathway, the size of the point indicates the number of genes enriched to the gene in each entry, and the significance of the point's enrichment.

© The Author(s)

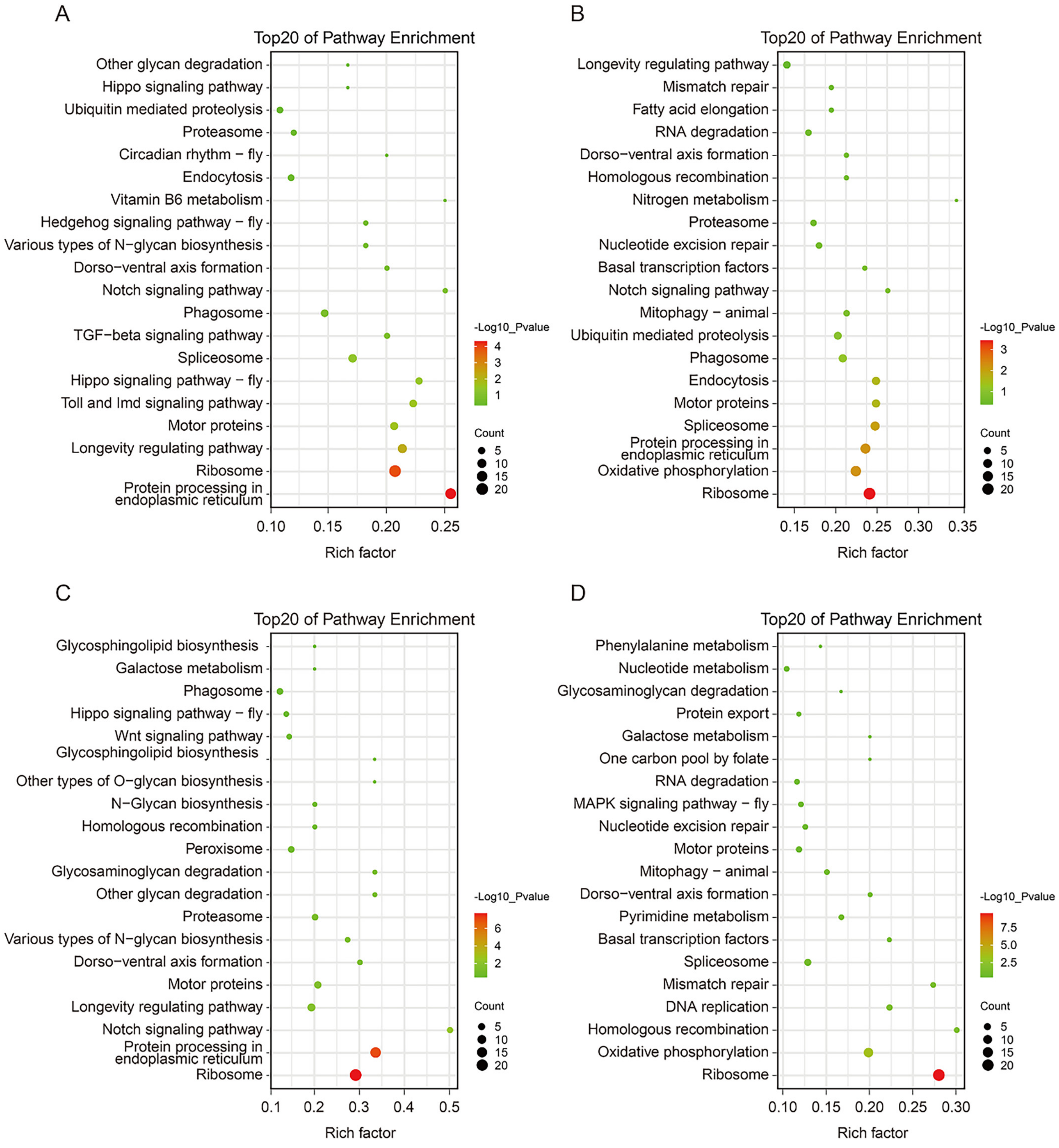

Figure EV8. Top 20 results of KEGG enrichment analysis of differentially expressed genes in four wing cell types of silkworm.

Vertical coordinate biological function of the gene or the pathway in which each molecule exerts its function, horizontal coordinate the percentage of the number of genes enriched to the target pathway, the size of the point indicates the number of genes enriched to the gene in each entry, and the significance of the point's enrichment.

 © The Author(s)

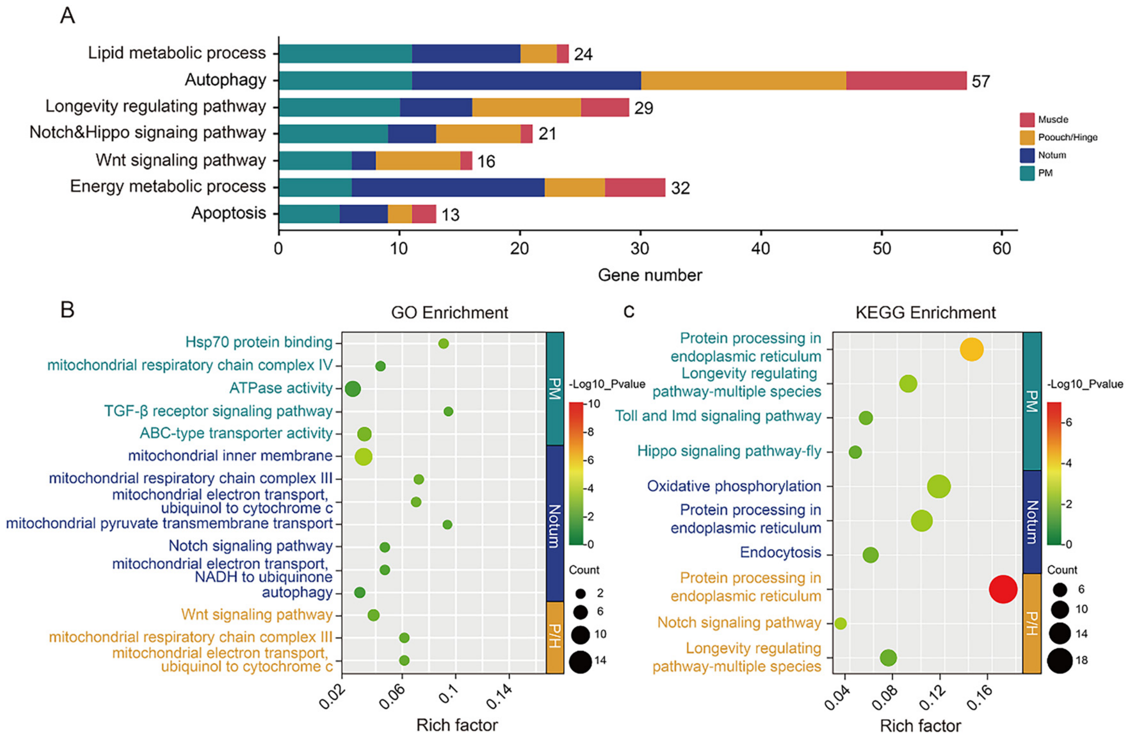

Figure EV9. Statistical analysis of differentially expressed genes in three wing cell types of the silkworm.

(**A**) Bar plot showing the number of differentially expressed genes (DEGs) associated with specific pathways. The x-axis represents the number of genes, and the y-axis lists the pathways: Lipid metabolic process (lipid metabolism-related genes), Autophagy (autophagy regulation-related genes), Longevity regulating pathway (lifespan regulation-related genes), Notch & Hippo signaling pathway (Notch and Hippo pathway-related genes), Wnt signaling pathway (Wnt pathway-related genes), Energy metabolic process (energy metabolism-related genes), and Apoptosis (apoptosis regulation-related genes). (**B**, **C**) Partial GO and KEGG enrichment analysis of DEGs in three wing cell types of silkworm. The top 20 enriched terms related to lipid metabolism, autophagy, energy metabolism, wing development, and lifespan regulation are displayed. The y-axis represents the biological functions or pathways, and the x-axis represents the percentage of genes enriched in each pathway. Dot size indicates the number of genes enriched in each term, and color intensity reflects the significance of enrichment.

© The Author(s)

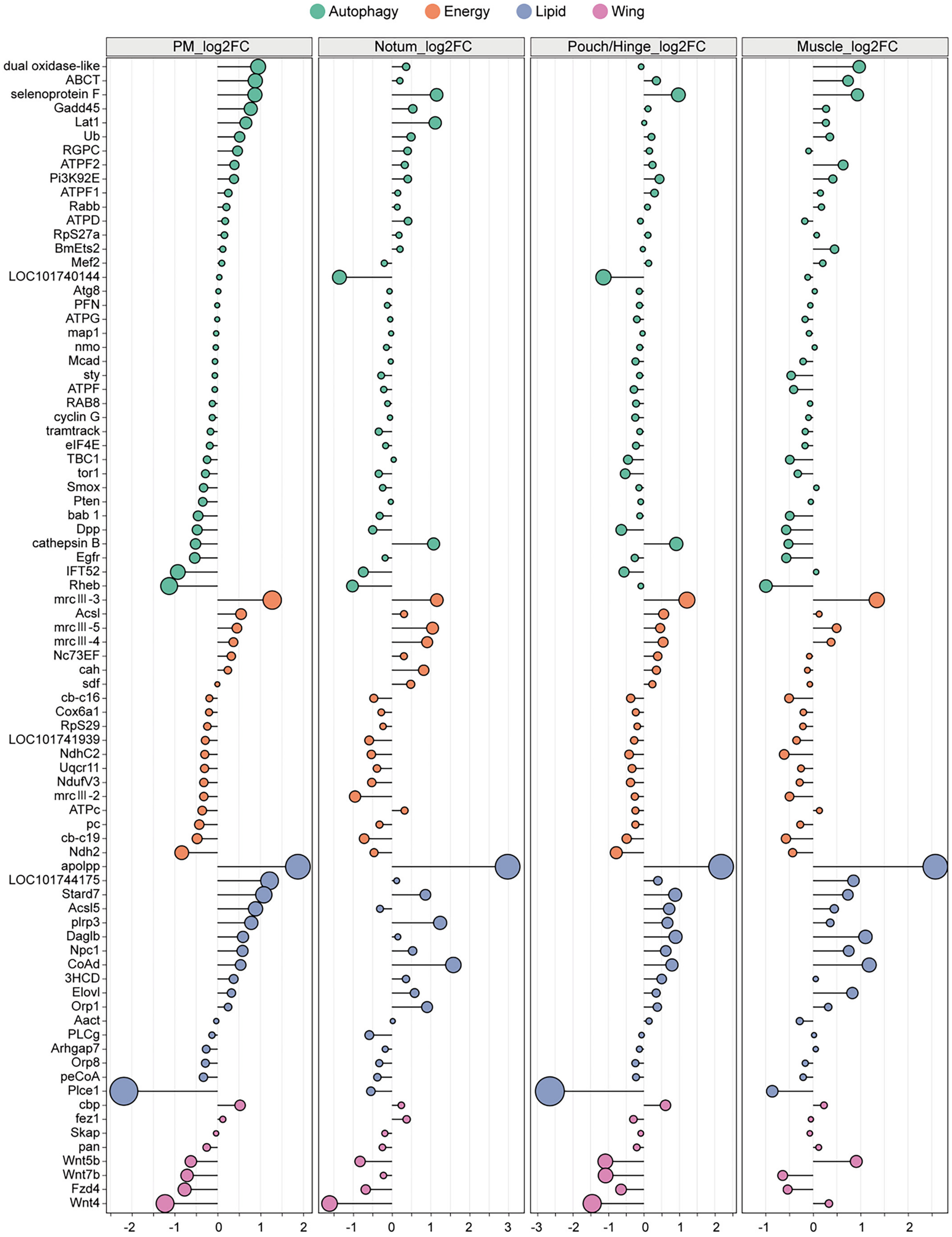
Autophagy
Energy
Lipid
Wing
PM_log2FC
Notum_log2FC
Pouch/Hinge_log2FC
Muscle_log2FC
dual oxidase-like
ABCT
selenoprotein F
Gadd45
Lat1
Ub
RGPC
ATPF2
Pi3K92E
ATPF1
Rabb
ATPD
RpS27a
BmEts2
Mef2
LOC101740144
Atg8
PFN
ATPG
map1
nmo
Mcad
sty
ATPF
RAB8
cyclin G
tramtrack
eIF4E
TBC1
tor1
Smox
Pten
bab 1
Dpp
cathepsin B
Egfr
IFT52
Rheb
mrcIII-3
Acsl
mrcIII-5
mrcIII-4
Nc73EF
cah
sdf
cb-c16
Cox6a1
RpS29
LOC101741939
NdhC2
Uqcr11
NdufV3
mrcIII-2
ATPc
pc
cb-c19
Ndh2
apolpp
LOC101744175
Stard7
Acsl5
plrp3
Daglb
Npc1
CoAd
3HCD
Elovl
Orp1
Aact
PLCg
Arhgap7
Orp8
peCoA
Plce1
cbp
fez1
Skap
pan
Wnt5b
Wnt7b
Fzd4
Wnt4

 © The Author(s)

Figure EV10. Comparison of lipid, energy, autophagy and wing development-related differential gene expression in four wing cell types of silkworm.

Vertical coordinates are the different differential genes, and horizontal coordinates are Log_2FC values; $Log_2FC > 0$, upregulates gene expression; $Log_2FC < 0$, downregulates gene expression.

© The Author(s)

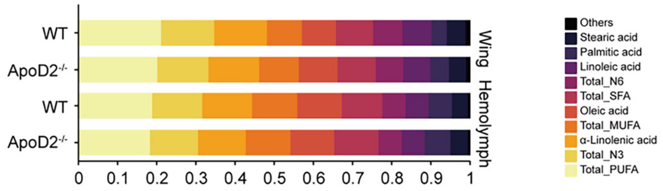

Figure EV11. Composition of fatty acids in the wing and hemolymph. Composition of fatty acids in the wing and hemolymph.

Stacked bar plots showing the relative proportions of major fatty acid species in the wing and hemolymph of WT and *ApoD2*$^{-/-}$ mutants. Each color represents a different fatty acid class as indicated in the legend.

 © The Author(s)
