# Supplementary material for: Apolipoprotein D neofunctionalization couples lipid allocation to wing evolution
Source: EMBO J. 2026 Jun 3;45(14):4956–87. doi: 10.1038/s44318-026-00821-0 (PMC13373240; doi:10.1038/s44318-026-00821-0)
Supplement: Supplementary file 1 — Peer Review File [file 44318_2026_821_MOESM1_ESM.pdf]

THE EMBO JOURNAL

Apolipoprotein D neofunctionalization couples lipid allocation to wing evolution

Yuxin Huang, Yinghui Li, Shunze Jia, Yanting Liang, Qingyu Lu, Wenhui Jing, and Huabing Wang

Corresponding author(s): Huabing Wang (wanghb@zju.edu.cn)

Review Timeline:

Submission Date:	6th Dec 25
Editorial Decision:	12th Feb 26
Revision Received:	20th Mar 26
Editorial Decision:	24th Apr 26
Revision Received:	2nd May 26
Accepted:	7th May 26

Editor: Yehu Moran

Transaction Report:

(Note: With the exception of the correction of typographical or spelling errors that could be a source of ambiguity, letters and reports are not edited. Depending on transfer agreements, referee reports obtained elsewhere may or may not be included in this compilation. Referee reports are anonymous unless the Referee chooses to sign their reports.)

Dear Prof. Wang,

Thank you for submitting your manuscript for consideration by the EMBO Journal. It has now been seen by two referees whose comments are shown below.

Given the referees' positive recommendations, I would like to invite you to submit a revised version of the manuscript, addressing thoroughly the comments of both reviewers. I should add that it is EMBO Journal policy to allow only a single round of major revision, and acceptance of your manuscript will therefore depend on the completeness of your responses in this revised version.

When preparing your letter of response to the referees' comments, please bear in mind that this will form part of the Review Process File, and will therefore be available online to the community. For more details on our Transparent Editorial Process, please review our Editorial Policies: https://link.springer.com/partners/embo-press/editorial-policies

We generally allow three months as standard revision time. As a matter of policy, competing manuscripts published during this period will not negatively impact on our assessment of the conceptual advance presented by your study. However, we request that you contact the editor as soon as possible upon publication of any related work, to discuss how to proceed. Should you foresee a problem in meeting this three-month deadline, please let us know in advance and we may be able to grant an extension.

Thank you for the opportunity to consider your work for publication. I look forward to your revision.

Yours sincerely,

Yehu Moran
Academic Editor
The EMBO Journal

Instructions for preparing your revised manuscript:

Read our guidance for manuscript revisions and related editorial policies: https://link.springer.com/journal/44318/submission-guidelines#cms-Revised-submissions

Please make sure you upload a letter of response to the referees' comments together with the revised manuscript.

Please also check that the title and abstract of the manuscript are brief, yet explicit, even to non-specialists.

When assembling figures, please refer to our figure preparation guideline in order to ensure proper formatting and readability in print as well as on screen:
https://media.springernature.com/original/springer-cms/rest/v1/content/27825798/data/v1

At EMBO Press we ask authors to provide source data for the main manuscript figures. You will receive a separate email with instructions for providing source data with your revised manuscript, including how to upload and organize the files.

IMPORTANT: When you send the revision we will require
- a point-by-point response to the referees' comments, with a detailed description of the changes made (as a word file).
- a word file of the manuscript text.
- individual production quality figure files (one file per figure)
- a complete author checklist
- Expanded View files (replacing Supplementary Information)
- a Reagents and Tools Table as part of the Methods section

Please remember: Digital image enhancement is acceptable practice, as long as it accurately represents the original data and conforms to community standards. If a figure has been subjected to significant electronic manipulation, this must be noted in the figure legend or in the 'Materials and Methods' section. The editors reserve the right to request original versions of figures and the original images that were used to assemble the figure.

We realize that it is difficult to revise to a specific deadline. In the interest of protecting the conceptual advance provided by the work, we recommend a revision within 3 months (13th May 2026). Please discuss the revision progress ahead of this time with the editor if you require more time to complete the revisions. Use the link below to submit your revision:

https://emboj.msubmit.net/cgi-bin/main.plex

Referee #1:

The authors performed comparative single-cell RNA-seq (scRNA-seq) analysis and comparative metabolomic/lipidomic analyses between WT and a BmApoD2 KO mutant in Bombyx mori. As a result, they found that the fatty acid metabolism is repressed and energy production is also reduced in BmApoD2 KO. In addition, the authors found that cell composition (cell numbers per cell type) changed dramatically in pupal wing discs, as revealed by scRNA-seq data. These results can explain the BmApoD2 KO phenotype (degenerative wing development) well. Their findings indicate that ApoD2 plays an important role in cellular metabolism and wing development in lepidopteran insects.

Although the author's group already published the phenotypes (including comparative RNA-seq at the individual level (not scRNA-seq)) of BmApoD2 KO (Jia et al., 2024), the current manuscript describes newer and much deeper findings at the single-cell level and reveals new insight about wing development.

My major criticism (question) is about the molecular function of the ApoD2 protein. In insects, the fatty acids are transported as diglyceride in the hemolymph with the help of lipophorin. How does ApoD2 work to transfer the lipids to wing disc cells? Neither the previous paper (Jia et al., 2024) nor the present manuscript discusses this important point.

My minor comments are as follows:

(1) Supplementary Figure 2. Did the authors obtain the CRISPR-based BmApoD2 KO line independently of the previous study (Jia et al., 2024)? In the "M1" KO line described in the previous paper, five nucleotides ACCCG were deleted. In the Supplementary Figure 2 of the present manuscript, the nucleotides GACCC were deleted (only one nucleotide shifted). Is it a newly obtained mutant?

(2) X and Y axes in PCA graphs in Figure 3 (C, D, G). "UAMP_1" and "UAMP_2" may be "UMAP_1" and "UMAP_2"?

(3) EM photograph in Figure 7C. The author should indicate the portion of the wing discs for the ultrathin-section preparation. Are the same portions of the wing compared? If possible, it should be correlated to the wing cell type described in Figures 3 and 4.

(4) Line 155 "associated with autophagy and energy metabolic processes in all three cell types". Indicate the three cell types.

(5) The authors use the term "peripodial membrane" in the main text. On the other hand, they describe "peripodial epithelium" in the legend to Figure 3. If they mean the same, the figure legend should be changed to "peripodial membrane".

Referee #2:

This manuscript by Huang and colleages investigates the neofunctionalization of Apolipoprotein D2 (ApoD2) in Lepidoptera. Using a comparative genomics approach, the authors discover that ApoD2 represents a duplication of ApoD, suggesting a novel function for this gene. Indeed, the authros generate a null allele of ApoD2 and discover that these animals exhibit a wing defect. Subsequent transcriptomic and metabolic analysis reveal that that loss of ApoD2 disrupts mitochondrial function, impairs β oxidation, alters epithelial and muscle cell homeostasis. Moreover, lipidomic analysis hints at major defects in lipid trafficking to the developing with. Overall, I find this to be a fascinating and important study. I think this will be a highly cited study - this manuscript could cement Bombyx as the go to study for studying lipid trafficking in insect - displacing Drosophila from this role. To cement the prominence of this study, I would ask for the following revisions.

1. The phenotype of ApoD2 mutants looks quite familiar to me - it seems to phenocopy the wing defects of Drosophila melanogaster withered mutants. Since withered encodes CPTI, the current study highlights the conserved requirement for lipid dependent wing development. I would insist that the authors discuss these D. melanogaster mutants as it adds further value to their study. Studies of withered highlight cell autonomous mechanisms - the current study discovers mechanisms of lipid trafficking in wing development. In fact, as a fly geneticists, I walked away from this study thinking that Bombyx might be the superior model for studying this phenomenon. Thus, discussing the fly studies would enhance the overall impact of the

manuscript.

2. The description of the lipidomics methodology is inadequate. I would recommend that the authors follow the guidelines for lipidomic studies required by the Journal of Lipid Research (see here: https://www.jlr.org/content/authorinfo - see Lipidomics section). As written, I'm uncertain as to the quality of the lipidomics data.

3. Related to point 2, further details are required for the targeted metabolomics methodology. How were metabolites extracted, derivatized, ect? The methods should provide the details required to repeat this analysis.

4. In general, the figure legends need additional details. For example, Figure 6A lacks any details related to statistical analysis or sample number. Please provide all necessary details regarding statistical analysis.

5. The text occasionally makes statement that are not supported by the results. For example, in line 161, the authors stat the "Its loss triggers a collaps of mitochondrial respiration and a shift toward lipid synthesis..." This statement is not supported in the text and would require respirometry or flux analysis. If the authors wish to make these statements, they need to provide additional experimentation. My suggestion, however, would be to simply temper the text to accurately reflect the results. Similar occurrences can be found throughout.

Point-by-point responses to two nice reviewers are listed below.

Referee #1:

The authors performed comparative single-cell RNA-seq (scRNA-seq) analysis and comparative metabolomic/lipidomic analyses between WT and a BmApoD2 KO mutant in *Bombyx mori*. As a result, they found that the fatty acid metabolism is repressed and energy production is also reduced in BmApoD2 KO. In addition, the authors found that cell composition (cell numbers per cell type) changed dramatically in pupal wing discs, as revealed by scRNA-seq data. These results can explain the BmApoD2 KO phenotype (degenerative wing development) well. Their findings indicate that ApoD2 plays an important role in cellular metabolism and wing development in lepidopteran insects.

Although the author's group already published the phenotypes (including comparative RNA-seq at the individual level (not scRNA-seq)) of BmApoD2 KO (Jia et al., 2024), the current manuscript describes newer and much deeper findings at the single-cell level and reveals new insight about wing development.

Response:

Thank you very much for your excellent suggestions and positive evaluation. Those comments and suggestions are very helpful for improving our paper. We're grateful for your thorough review and have carefully considered each of your comments and suggestions. We revised the manuscript point by point.

My major criticism (question) is about the molecular function of the ApoD2 protein. In insects, the fatty acids are transported as diglyceride in the hemolymph with the help of lipophorin. How does ApoD2 work to transfer the lipids to wing disc cells? Neither the previous paper (Jia et al., 2024) nor the present manuscript discusses this important point.

Response:

Thank you very much for raising this important mechanistic question. We agree that clarifying how ApoD2 participates in lipid delivery to wing disc cells within the canonical lipophorin-mediated lipid transport system is essential. In insects, circulating lipids are transported predominantly as diacylglycerol (DAG) by lipophorin particles between the fat body and peripheral tissues (Arrese & Soulages, 2010). ApoD2, as a secreted lipocalin carrying a conserved calyx-shaped hydrophobic binding pocket with

demonstrated affinity for fatty acids and signaling lipids such as arachidonic acid (Kielkopf *et al*, 2018; Marques & Gallazzini, 2024; Sanchez & Ganfornina, 2021). By analogy with vertebrate ApoD, which associates predominantly with high-density lipoprotein (HDL) particles, ApoD2 may associate with circulating lipophorin particles via hydrophobic surface patches on its structure. Our lipidomic data reveal a lipid redistribution phenotype: hemolymph DAG levels are largely unaffected in *BmApoD2* mutants, whereas DAG and TAG are selectively depleted in the wing. These results suggest that ApoD2 functions not as a bulk lipid carrier competing with lipophorin, but as a selective lipid transfer protein operating at the interface between hemolymph circulation and wing disc uptake to regulate lipid acquisition and distribution in the wing disc. We have accordingly expanded the discussion of this model (lines 300–312). We have updated the graphical abstract figure. Elucidating the receptor responsible for ApoD2 recognition and directly visualizing the ApoD2-mediated lipid transfer process will represent important priorities for future investigation.

My minor comments are as follows:

(1) Supplementary Figure 2. Did the authors obtain the CRISPR-based BmApoD2 KO line independently of the previous study (Jia et al., 2024)? In the "M1" KO line described in the previous paper, five nucleotides ACCCG were deleted. In the Supplementary Figure 2 of the present manuscript, the nucleotides GACCC were deleted (only one nucleotide shifted). Is it a newly obtained mutant?

Response:

We thank the referee for carefully noting this detail. The ApoD2-null line used in this study corresponds to the same M1 allele previously reported (Jia et al., 2024), in which deletion of five nucleotides (ACCCG) results in a frameshift mutation. The sequence shown in Supplementary Figure 2 of the originally submitted version (GACCC) was a typographical annotation error introduced during figure preparation. We have corrected the sequence in Supplementary Figure 2 to ACCCG. We apologize for this oversight and thank the referee for bringing it to our attention.

(2) X and Y axes in PCA graphs in Figure 3 (C, D, G). "UAMP_1" and "UAMP_2" may be "UMAP_1" and "UMAP_2"?

Response: We thank the referee for carefully examining this detail. We corrected it in Figure 3.

(3) EM photograph in Figure 7C. The author should indicate the portion of the wing discs for the ultrathin-section preparation. Are the same portions of the wing compared? If possible, it should be correlated to the wing cell type described in Figures 3 and 4.

Response: We thank the reviewer for this valuable suggestion. For the ultrathin-section preparation in Figure 7C, sections were taken from the total wing disc of both wild-type and *ApoD2*$^{-/-}$ mutants. During the transmission electron microscopy analyses, despite multiple attempts, it was difficult to capture regions corresponding to the same cell type. In the future, we plan to utilize spatial omics approaches to conduct comparative analyses of identical regions. We acknowledge that without spatial transcriptomic data, we are currently unable to unambiguously assign the ultrastructural observations to defined cell populations, and cannot fully exclude the possibility that sampling from different regions of the wing disc may have introduced bias into the results. To address this concern and avoid potential misinterpretation, we have therefore removed Figure 7C (TEM data) from the manuscript.

(4) Line 155 "associated with autophagy and energy metabolic processes in all three cell types". Indicate the three cell types.

Response:

Thank you very much for pointing out this ambiguity. The three cell types referred to are the three wing cell subtypes identified in the pupal wing: peripodial membrane (PM), notum, and pouch/hinge cells. We have revised the text accordingly to explicitly name these cell types. These changes can be found on lines 154–155.

(5) The authors use the term "peripodial membrane" in the main text. On the other hand, they describe "peripodial epithelium" in the legend to Figure 3. If they mean the same, the figure legend should be changed to "peripodial membrane".

Response:

We thank the reviewer for identifying this inconsistency. "Peripodial membrane" and "peripodial epithelium" were used interchangeably in the manuscript, which is indeed inconsistent. We have standardized the terminology to "peripodial membrane" throughout the manuscript, including the legend to Figure 3.

Thank you very much again for reviewing our manuscript again.

Referee #2:

This manuscript by Huang and colleages investigates the neofunctionalization of Apolipoprotein D2 (ApoD2) in Lepidoptera. Using a comparative genomics approach, the authors discover that ApoD2 represents a duplication of ApoD, suggesting a novel function for this gene. Indeed, the authros generate a null allele of ApoD2 and discover that these animals exhibit a wing defect. Subsequent transcriptomic and metabolic analysis reveal that that loss of ApoD2 disrupts mitochondrial function, impairs β oxidation, alters epithelial and muscle cell homeostasis. Moreover, lipidomic analysis hints at major defects in lipid trafficking to the developing with. Overall, I find this to be a fascinating and important study. I think this will be a highly cited study - this manuscript could cement Bombyx as the go to study for studying lipid trafficking in insect - displacing Drosophila from this role. To cement the prominence of this study, I would ask for the following revisions.

Response:

Thanks a lot for your excellent suggestion and positive evaluation. Those comments and suggestions are helpful for improving our paper. We're grateful for your thorough review and have carefully considered each of your comments and suggestions. We revised the manuscript point by point.

1. The phenotype of ApoD2 mutants looks quite familiar to me - it seems to phenocopy the wing defects of *Drosophila melanogaster* withered mutants. Since withered encodes CPTI, the current study highlights the conserved requirement for lipid dependent wing development. I would insist that the authors discuss these *D. melanogaster* mutants as it adds further value to their study. Studies of withered highlight cell autonomous mechanisms - the current study discovers mechanisms of lipid trafficking in wing development. In fact, as a fly geneticists, I walked away from this study thinking that *Bombyx* might be the superior model for studying this phenomenon. Thus, discussing the fly studies would enhance the overall impact of the manuscript.

Response:

Thank you very much for your valuable suggestion. We thank the reviewer for drawing our attention to the parallel between ApoD2 mutants and *Drosophila melanogaster* withered (*whd*) mutants. We agree that discussing these studies substantially strengthens the evolutionary and mechanistic framework of our manuscript. We have

incorporated a discussion of these findings into the revised manuscript (lines 320–329), highlighting the phenotypic parallels between the two models and contrasting the cell-autonomous lipid metabolic defects revealed by studies on *whd* mutants in *D. melanogaster* with the inter-tissue lipid trafficking dysfunction uncovered in the present study. We concur with the reviewer's assessment that *B. mori* offers complementary advantages for dissecting the full spectrum of lipid-dependent mechanisms underlying wing morphogenesis.

2. The description of the lipidomics methodology is inadequate. I would recommend that the authors follow the guidelines for lipidomic studies required by the Journal of Lipid Research (see here: https://www.jlr.org/content/authorinfo - see Lipidomics section). As written, I'm uncertain as to the quality of the lipidomics data.

Response:

According to your valuable suggestion, we revised the lipidomics methodology to comply with the reporting guidelines recommended by the Journal of Lipid Research. The revised methodology now includes: (1) deuterium-labeled internal standards for each major lipid class to monitor extraction recovery; (2) full MS/MS acquisition parameters enabling structural-level lipid identification; (3) retention of only grade A–B identifications with ≤5 ppm mass accuracy; (4) QC-based batch correction with a CV threshold of <30%; and (5) normalization to both internal standards and tissue weight. Together, these measures ensure the accuracy, reproducibility, and structural reliability of the lipidomic dataset. These changes can be found on lines 431–455.

3. Related to point 2, further details are required for the targeted metabolomics methodology. How were metabolites extracted, derivatized, ect? The methods should provide the details required to repeat this analysis.

Response:

Thank you very much for your nice suggestion. We have substantially expanded the targeted metabolomics methodology to provide sufficient detail for reproducibility. The revised section now includes: (1) full sample preparation and extraction procedures; (2) acid-catalyzed transmethylation conditions for FAME derivatization; (3) complete GC-MS instrument parameters; and (4) quantification details including isotope dilution against external calibration curves with normalization to tissue input weight (lines 458–476).

4. In general, the figure legends need additional details. For example, Figure 6A lacks any details related to statistical analysis or sample number. Please provide all necessary details regarding statistical analysis.

Response:

We thank the reviewer for this helpful suggestion. Following the reviewer's recommendation, we have revised all figure legends to include complete statistical information, including sample sizes, number of biological replicates, statistical tests used, and significance thresholds. Specifically, Figures 6A and 6D now report the sample numbers, mean ± SEM, and the statistical methods applied. Figure 7A has been updated in the same manner. These additions ensure that all statistical analyses are clearly documented in the figure legends.

5. The text occasionally makes statement that are not supported by the results. For example, in line 161, the authors stat the "Its loss triggers a collaps of mitochondrial respiration and a shift toward lipid synthesis..." This statement is not supported in the text and would require respirometry or flux analysis. If the authors wish to make these statements, they need to provide additional experimentation. My suggestion, however, would be to simply temper the text to accurately reflect the results. Similar occurrences can be found throughout.

Response:

We thank the reviewer for this important and constructive comment. We agree that several statements in the original manuscript overstated the conclusions beyond what the experimental data directly support. We have carefully revised the text throughout the manuscript to ensure that all statements are appropriately tempered and accurately reflect our experimental results. The relevant revisions can be found on lines 151–152, 159–162, 176–177, 185–190, 238–242, 248–250, 256–257, 262–267, 282–285, 292–294, 317–319, and 332–335.

Thank you very much again for reviewing our manuscript again.

Reference

Arrese EL, Soulages JL (2010). Insect Fat Body: Energy, Metabolism, and Regulation. Annu Rev Entomol 55: 207-225.

Kielkopf CS, Low JKK, Mok YF, Bhatia S, Palasovski T, Oakley AJ, Whitten AE, Garner B, Brown SHJ (2018). Identification of a novel tetrameric structure for human apolipoprotein-D. J Struct Biol 203: 205-218.

Marques E, Gallazzini M (2024). The Lipocalins. Curr Biol 34: R670-R672.

Sanchez D, Ganfornina MD (2021). The Lipocalin Apolipoprotein D Functional Portrait: A Systematic Review. Front Physiol 12: 738991.

Dear Prof. Wang,

Thank you for submitting your manuscript for consideration by the EMBO Journal. It has now been seen by two of the orignal referees whose comments are enclosed. As you will see, borh express interest in your manuscript and are broadly in favour of publication. Yet, Referee #1 still has minor comments that need your attention. Moreover, our editorial assistance team has flagged several technical issues that must be corrected before your paper can be accepted by the EMBO Journal.

Please revise the manuscript to address these issues and submit the revised version at your earliest convenience.

When preparing your letter of response to the referees' comments, please bear in mind that this will form part of the Review Process File, and will therefore be available online to the community. For more details on our Transparent Editorial Process, please review our Editorial Policies page: https://link.springer.com/partners/embo-press/editorial-policies

We generally allow three months as standard revision time. As a matter of policy, competing manuscripts published during this period will not negatively impact on our assessment of the conceptual advance presented by your study. However, we request that you contact the editor as soon as possible upon publication of any related work, to discuss how to proceed.

Thank you for the opportunity to consider your work for publication. I look forward to your revision.

Yours sincerely,

Yehu Moran
Academic Editor
The EMBO Journal

Instructions for preparing your revised manuscript:

Read our guidance for manuscript revisions and related editorial policies: https://link.springer.com/journal/44318/submission-guidelines#cms-Revised-submissions

Please also check that the title and abstract of the manuscript are brief, yet explicit, even to non-specialists.

When assembling figures, please refer to our figure preparation guideline in order to ensure proper formatting and readability in print as well as on screen:
https://media.springernature.com/original/springer-cms/rest/v1/content/27825798/data/v1

IMPORTANT: When you send the revision we will require
- a point-by-point response to the referees' comments, with a detailed description of the changes made (as a word file).
- a word file of the manuscript text.
- individual production quality figure files (one file per figure)
- a complete author checklist
- Expanded View files (replacing Supplementary Information)
- a Reagents and Tools Table as part of the Methods section

Please remember: Digital image enhancement is acceptable practice, as long as it accurately represents the original data and conforms to community standards. If a figure has been subjected to significant electronic manipulation, this must be noted in the figure legend or in the 'Methods' section. The editors reserve the right to request original versions of figures and the original images that were used to assemble the figure.

We realize that it is difficult to revise to a specific deadline. In the interest of protecting the conceptual advance provided by the work, we recommend a revision within 3 months (23rd Jul 2026). Please discuss the revision progress ahead of this time with the editor if you require more time to complete the revisions. Use the link below to submit your revision:

https://emboj.msubmit.net/cgi-bin/main.plex

----

comments by editorial assistance team

- FIGURES IN SEPARATE FILES: Please remove the figures from the manuscript text file. Please rename the supplementary figures "Figure EV1" etc. and upload them as separate, high resolution figure files. Add the figure legends to the manuscript text, after the main figure legends and under the heading "Expanded View Figure Legends".
- DISCLOSURE AND COMPETING INTERESTS STATEMENT: Please add a Disclosure and Competing Interests Statement to the manuscript text, after the Acknowledgments
- DATASET EV LEGENDS: Please upload the supplementary tables as one file per table and rename them "Dataset EV1" - "Dataset EV14". Please make sure to update their callouts in the manuscript text.
- REAGENT TABLE: Thank you for providing a reagents and tools table. Please remove it from the manuscript text and upload it as a separate file.
- SYNOPSIS IMAGE: Thank you for uploading a synopsis image. Please note that we require this in the exact dimensions 550 pixels wide x 300 pixels high. Please reformat the resize the image to those dimensions.
- SYNOPSIS TEXT: Please upload a word file with the synopsis, consisting of a summary of the study and 3 to five bullet points with the most important findings. Please look on our website for examples from recently accepted papers.

Figure 7B - LKB1 blot is compressed vertically. There also appears to be some editing of the background in comparison to the source data. Please remake the figure using the unaltered captured source data.

1. Please note that the specific URLs for MTBLS11285, PRJCA060360 datasets are not provided in the data availability statement. This should be corrected and they should be publicly available upon acceptance.

- Figure legends:
1. Please define the annotated p values ****/***/**/* as well as provide the exact p-values for the same in the legend of figure 4C as appropriate.
2. Please note that the exact p values are not provided in the legends of figures 4C, 6A, C; 7A
3. Please indicate the statistical test used for data analysis in the legends of figure 4C
4. Please note that the box plots need to be defined in terms of minima, maxima, centre, bounds of box and whiskers, and percentile in the legends of figures 4C, 6C
5. Please note that information related to n is missing in the legends of figures 4C, 5D, F, G

comments by Referees
Referee #1:

The authors have adequately revised the manuscript in response to my comments, particularly regarding the mechanistic aspect of the ApoD2 protein in lipid transfer from the hemolymph to wing disks, which was my concern.
Although I did not expect the electron microscopy data to be removed, the authors' decision seems reasonable.
Overall, the manuscript has improved considerably.
I have noticed the following minor mistakes, which should be corrected during the re-revision process, though I did not notice some of them in my first-round review (some of them are new in the revised manuscript).

line 65
(Wootton & KukalovÃ-Peck 2000)
should be corrected to
(Wootton & Kukalová-Peck 2000).

line 603
Drosophila melanogaster
should be italicized.

line 609
Wootton RJ, KukalovÃ-Peck J.
should be corrected to
Wootton RJ, Kukalová-Peck J.

Supplementary table1
line 2
"Order" should be changed to "Class" because all of the descriptions in the Class row are Classes in taxonomy.

Referee #2:

The authors have addressed my concerns.

Comments by editorial assistance team

- FIGURES IN SEPARATE FILES: Please remove the figures from the manuscript text file. Please rename the supplementary figures "Figure EV1" etc. and upload them as separate, high resolution figure files. Add the figure legends to the manuscript text, after the main figure legends and under the heading "Expanded View Figure Legends".

Response: All figures have been removed from the manuscript text file and uploaded as separate high-resolution files. Supplementary figures have been renamed as Figure EV1, EV2, etc. Figure legends for Expanded View figures have been added to the manuscript text under the heading “Expanded View Figure Legends.”

- DISCLOSURE AND COMPETING INTERESTS STATEMENT: Please add a Disclosure and Competing Interests Statement to the manuscript text, after the Acknowledgments

Response: A Disclosure and Competing Interests Statement has been added after the Acknowledgments section. The authors declare no competing interests.

- DATASET EV LEGENDS: Please upload the supplementary tables as one file per table and rename them "Dataset EV1" - "Dataset EV14". Please make sure to update their callouts in the manuscript text.

Response: All supplementary tables have been uploaded as individual files and renamed as Dataset EV1 through Dataset EV14. All callouts in the manuscript text have been updated accordingly.

- REAGENT TABLE: Thank you for providing a reagents and tools table. Please remove it from the manuscript text and upload it as a separate file.

Response: The Reagents and Tools Table has been removed from the manuscript text and uploaded as a separate file.

- SYNOPSIS IMAGE: Thank you for uploading a synopsis image. Please note that we

require this in the exact dimensions 550 pixels wide x 300 pixels high. Please reformat the resize the image to those dimensions.

Response: The synopsis image has been reformatted to the required dimensions of 550 x 300 pixels.

- SYNOPSIS TEXT: Please upload a word file with the synopsis, consisting of a summary of the study and 3 to five bullet points with the most important findings. Please look on our website for examples from recently accepted papers.

Response: A Word file containing the synopsis summary and key findings has been prepared and uploaded.

Figure 7B - LKB1 blot is compressed vertically. There also appears to be some editing of the background in comparison to the source data. Please remake the figure using the unaltered captured source data.

Response: Figure 7B has been remade using the original, unaltered source data.

1. Please note that the specific URLs for MTBLS11285, PRJCA060360 datasets are not provided in the data availability statement. This should be corrected and they should be publicly available upon acceptance.

Response: The specific URLs for both datasets (MTBLS11285 and PRJCA060360) have been added to the Data Availability Statement (lines 475-479).

- Figure legends:

1. Please define the annotated p values ****/***/**/* as well as provide the exact p-values for the same in the legend of figure 4C as appropriate.

Response: The p-value annotations (*p < 0.05, **p < 0.01, ***p < 0.001) have been defined, and exact p-values for all nine metabolic pathways have been provided in the revised Figure 4 legend (lines 730–735).

2. Please note that the exact p values are not provided in the legends of figures 4C, 6A,

C; 7A

Response: Exact p-values have been added to the "Data information" sections in the revised legends of Figures 4C (lines 730–735), 6A and 6C (lines 771–783), and 7A (lines 791–796).

3. Please indicate the statistical test used for data analysis in the legends of figure 4C

Response: The statistical test (two-sided Wilcoxon rank-sum test) has been specified in the revised Figure 4 legend (lines 729–730).

4. Please note that the box plots need to be defined in terms of minima, maxima, centre, bounds of box and whiskers, and percentile in the legends of figures 4C, 6C

Response: Box plot definitions have been added to the revised legends of Figures 4C (lines 724–727) and 6C (lines 762–765).

5. Please note that information related to n is missing in the legends of figures 4C, 5D, F, G

Response: n have been added to the revised legends of Figures 4C (lines 727–729) and 5D, F, G (lines 747–750).

comments by Referees

Referee #1:

The authors have adequately revised the manuscript in response to my comments, particularly regarding the mechanistic aspect of the ApoD2 protein in lipid transfer from the hemolymph to wing disks, which was my concern.

Although I did not expect the electron microscopy data to be removed, the authors' decision seems reasonable.

Overall, the manuscript has improved considerably.

I have noticed the following minor mistakes, which should be corrected during the re-revision process, though I did not notice some of them in my first-round review (some

of them are new in the revised manuscript).

We are grateful for your positive evaluation and excellent suggestions. Your comments are very helpful for revising and improving our paper. We revised the manuscript point by point.

line 65 (Wootton & KukalovÃ-Peck 2000) should be corrected to (Wootton & Kukalová-Peck 2000).

Response: Thank you very much for your valuable suggestion. The author name has been fixed to “Kukalová-Peck” with the correct diacritical mark.

line 603 Drosophila melanogaster should be italicized.

Response: Thank you very much for your nice suggestion. “*Drosophila melanogaster*” has been italicized on line 603.

line 609 Wootton RJ, KukalovÃ-Peck J. should be corrected to Wootton RJ, Kukalová-Peck J.

Response: We thank the reviewer for this helpful suggestion. The reference has been updated with the correct spelling “Kukalová-Peck J.”

Supplementary table1 line 2 "Order" should be changed to "Class" because all of the descriptions in the Class row are Classes in taxonomy.

Response: We thank the reviewer for this valuable suggestion. “Order” has been changed to “Class” in line 2 of Supplementary Table 1 (now Dataset EV1).

Thank you very much again for reviewing our manuscript.

Referee #2:

The authors have addressed my concerns.

Thank you very much again for reviewing our manuscript again.

Dear Prof. Wang,

I am pleased to inform you that your manuscript has been accepted for publication in the EMBO Journal.

Your manuscript will be processed for publication by EMBO Press. It will be copy edited and you will receive page proofs prior to publication. Please note that you will be contacted by Springer Nature Author Services to complete licensing and payment information.

You may qualify for financial assistance for your publication charges - either via a Springer Nature fully open access agreement or an EMBO initiative. Check your eligibility: https://link.springer.com/journal/44318/how-to-publish-with-us

Should you be planning a Press Release on your article, please get in contact with embo_production@springernature.com as early as possible in order to coordinate publication and release dates.

If you have any questions, please do not hesitate to contact the Editorial Office. Thank you for your contribution to The EMBO Journal.

Yours sincerely,

Yehu Moran
Academic Editor
The EMBO Journal

*****
Please note that it is The EMBO Journal policy for the transcript of the editorial process (containing referee reports and your response letters) to be published as an online supplement to each paper. If you should prefer removal of any referee-only figures included in the point-by-point response(s), e.g. because they may still be used for future publication or because they have been reproduced from published work by others, please do let us know immediately via response email.
More information is available here: https://link.springer.com/partners/embo-press/editorial-policies#Peer%20review

** Click here to be directed to your login page: https://emboj.msubmit.net

EMBO Press Author Checklist

Corresponding Author Name: HuabingWang	
Journal Submitted to: The EMBO journal	
Manuscript Number: EMBOJ-2025-123236R	

USEFUL LINKS FOR COMPLETING THIS FORM
The EMBO Journal - Author Guidelines
EMBO Reports - Author Guidelines
Molecular Systems Biology - Author Guidelines
EMBO Molecular Medicine - Author Guidelines

Reporting Checklist for Life Science Articles (updated January
This checklist is adapted from Materials Design Analysis Reporting (MDAR) Checklist for Authors. MDAR establishes a minimum set of requirements in transparent reporting in the life sciences (see Statement of Task: 10.31222/osf.io/9sm4x). Please follow the journal's guidelines in preparing your
Please note that a copy of this checklist will be published alongside your article.

Abridged guidelines for figures
1. Data
The data shown in figures should satisfy the following conditions:
- the data were obtained and processed according to the field's best practice and are presented to reflect the results of the experiments in an accurate and unbiased manner.
- ideally, figure panels should include only measurements that are directly comparable to each other and obtained with the same assay.
- plots include clearly labeled error bars for independent experiments and sample sizes. Unless justified, error bars should not be shown for technical
- if n<5, the individual data points from each experiment should be plotted. Any statistical test employed should be justified.
- Source Data should be included to report the data underlying figures according to the guidelines set out in the authorship guidelines on Data

2. Captions
Each figure caption should contain the following information, for each panel where they are relevant:
- a specification of the experimental system investigated (eg cell line, species name).
- the assay(s) and method(s) used to carry out the reported observations and measurements.
- an explicit mention of the biological and chemical entity(ies) that are being measured.
- an explicit mention of the biological and chemical entity(ies) that are altered/varied/perturbed in a controlled manner.
- the exact sample size (n) for each experimental group/condition, given as a number, not a range;
- a description of the sample collection allowing the reader to understand whether the samples represent technical or biological replicates (including how many animals, litters, cultures, etc.).
- a statement of how many times the experiment shown was independently replicated in the laboratory.
- definitions of statistical methods and measures:
  - common tests, such as t-test (please specify whether paired vs. unpaired), simple χ2 tests, Wilcoxon and Mann-Whitney tests, can be unambiguously identified by name only, but more complex techniques should be described in the methods section;
  - are tests one-sided or two-sided?
  - are there adjustments for multiple comparisons?
  - exact statistical test results, e.g., P values = x but not P values < x;
  - definition of 'center values' as median or average;
  - definition of error bars as s.d. or s.e.m.

Please complete ALL of the questions below.
Select "Not Applicable" only when the requested information is not relevant for your study.

Materials

Newly Created Materials	Information included in the manuscript?	In which section is the information available? (Reagents and Tools Table, Materials and Methods, Figures, Data Availability Section)
New materials and reagents need to be available; do any restrictions apply?	Yes	Reagents and Tools Table

Antibodies	Information included in the manuscript?	In which section is the information available? (Reagents and Tools Table, Materials and Methods, Figures, Data Availability Section)
For **antibodies** provide the following information:<br>- Commercial antibodies: RRID (if possible) or supplier name, catalogue number and or/clone number<br>- Non-commercial: RRID or citation	Yes	Reagents and Tools Table

DNA and RNA sequences	Information included in the manuscript?	In which section is the information available? (Reagents and Tools Table, Materials and Methods, Figures, Data Availability Section)
**Short novel DNA or RNA including primers, probes**: provide the sequences.	Not Applicable	

Cell materials	Information included in the manuscript?	In which section is the information available? (Reagents and Tools Table, Materials and Methods, Figures, Data Availability Section)
**Cell lines:** Provide species information, strain. Provide accession number in repository **OR** supplier name, catalog number, clone number, and/**OR** RRID.	Not Applicable	
**Primary cultures:** Provide species, strain, sex of origin, genetic modification status.	Not Applicable	
Report if the cell lines were recently **authenticated** (e.g., by STR profiling) and tested for mycoplasma contamination.	Not Applicable	

Experimental animals	Information included in the manuscript?	In which section is the information available? (Reagents and Tools Table, Materials and Methods, Figures, Data Availability Section)
**Laboratory animals or Model organisms:** Provide species, strain, sex, age, genetic modification status. Provide accession number in repository **OR** supplier name, catalog number, clone number, **OR** RRID.	Yes	Materials and Methods, Reagents and Tools Table
**Animal observed in or captured from the field:** Provide species, sex, and age where possible.	Not Applicable	
Please detail **housing and husbandry conditions**.	Yes	Materials and Methods

Plants and microbes	Information included in the manuscript?	In which section is the information available? (Reagents and Tools Table, Materials and Methods, Figures, Data Availability Section)
**Plants:** provide species and strain, ecotype and cultivar where relevant, unique accession number if available, and source (including location for collected wild specimens).	Not Applicable	
**Microbes:** provide species and strain, unique accession number if available, and source.	Not Applicable	

Human research participants	Information included in the manuscript?	In which section is the information available? (Reagents and Tools Table, Materials and Methods, Figures, Data Availability Section)
If collected and within the bounds of privacy constraints report on age, sex and gender or ethnicity for all study participants.	Not Applicable	

Core facilities	Information included in the manuscript?	In which section is the information available? (Reagents and Tools Table, Materials and Methods, Figures, Data Availability Section)
If your work benefited from core facilities, was their service mentioned in the acknowledgments section?	Not Applicable	

Design

Study protocol	Information included in the manuscript?	In which section is the information available? (Reagents and Tools Table, Materials and Methods, Figures, Data Availability Section)
If study protocol has been **pre-registered, provide DOI in the manuscript**. For clinical trials, provide the trial registration number **OR** cite DOI.	Not Applicable	
Report the **clinical trial registration number** (at ClinicalTrials.gov or equivalent), where applicable.	Not Applicable	

Laboratory protocol	Information included in the manuscript?	In which section is the information available? (Reagents and Tools Table, Materials and Methods, Figures, Data Availability Section)
Provide DOI OR other citation details if **external detailed step-by-step protocols** are available.	Yes	Materials and Methods

Experimental study design and statistics	Information included in the manuscript?	In which section is the information available? (Reagents and Tools Table, Materials and Methods, Figures, Data Availability Section)
Include a statement about **sample size** estimate even if no statistical methods were used.	Yes	Materials and Methods, Figures
Were any steps taken to minimize the effects of subjective bias when allocating animals/samples to treatment (e.g. **randomization procedure**)? If yes, have they been described?	Not Applicable	
Include a statement about **blinding** even if no blinding was done.	Not Applicable	
Describe **inclusion/exclusion criteria** if samples or animals were excluded from the analysis. Were the criteria pre-established?<br><br>If sample or data points were omitted from analysis, report if this was due to attrition or intentional exclusion and provide justification.	Yes	Materials and Methods
For every figure, are **statistical tests** justified as appropriate? Do the data meet the assumptions of the tests (e.g., normal distribution)? Describe any methods used to assess it. Is there an estimate of variation within each group of data? Is the variance similar between the groups that are being statistically compared?	Yes	Materials and Methods, Figures

Sample definition and in-laboratory replication	Information included in the manuscript?	In which section is the information available? (Reagents and Tools Table, Materials and Methods, Figures, Data Availability Section)
In the figure legends: state number of times the experiment was **replicated** in laboratory.	Yes	Figures
In the figure legends: define whether data describe **technical or biological replicates**.	Yes	Figures

Ethics

Ethics	Information included in the manuscript?	In which section is the information available? (Reagents and Tools Table, Materials and Methods, Figures, Data Availability Section)
Studies involving **human participants**: State details of **authority granting ethics approval** (IRB or equivalent committee(s), provide reference number for approval.	Not Applicable	
Studies involving **human participants**: Include a statement confirming that **informed consent** was obtained from all subjects and that the experiments conformed to the principles set out in the WMA Declaration of Helsinki and the Department of Health and Human Services Belmont Report.	Not Applicable	
Studies involving **human participants:** For publication of **patient photos**, include a statement confirming that consent to publish was obtained.	Not Applicable	
Studies involving experimental **animals**: State details of **authority granting ethics approval** (IRB or equivalent committee(s), provide reference number for approval. Include a statement of compliance with ethical regulations.	Not Applicable	
Studies involving **specimen and field samples:** State if relevant **permits** obtained, provide details of authority approving study; if none were required, explain why.	Not Applicable	

Dual Use Research of Concern (DURC)	Information included in the manuscript?	In which section is the information available? (Reagents and Tools Table, Materials and Methods, Figures, Data Availability Section)
Could your study fall under dual use research restrictions? Please check biosecurity documents and list of **select agents and toxins** (CDC): https://www.selectagents.gov/sat/list.htm	Not Applicable	
If you used a select agent, is the security level of the lab appropriate and reported in the manuscript?	Not Applicable	
If a study is subject to dual use research of concern regulations, is the name of the **authority granting approval and reference number** for the regulatory approval provided in the manuscript?	Not Applicable	

Reporting

The MDAR framework recommends adoption of discipline-specific guidelines, established and endorsed through community initiatives. Journals have their own policy about requiring specific guidelines and recommendations to complement MDAR.

Adherence to community standards	Information included in the manuscript?	In which section is the information available? (Reagents and Tools Table, Materials and Methods, Figures, Data Availability Section)
State if relevant guidelines or checklists (e.g., **ICMJE, MIBBI, ARRIVE, PRISMA**) have been followed or provided.	Not Applicable	
For **tumor marker prognostic studies,** we recommend that you follow the **REMARK** reporting guidelines (see link list at top right). See author guidelines, under 'Reporting Guidelines'. Please confirm you have followed these guidelines.	Not Applicable	
For **phase II and III randomized controlled trials**, please refer to the **CONSORT** flow diagram (see link list at top right) and submit the CONSORT checklist (see link list at top right) with your submission. See author guidelines, under 'Reporting Guidelines'. Please confirm you have submitted this list.	Not Applicable	

Data Availability

Data availability	Information included in the manuscript?	In which section is the information available? (Reagents and Tools Table, Materials and Methods, Figures, Data Availability Section)
Have **primary datasets** been deposited according to the journal's guidelines (see 'Data Deposition' section) and the respective accession numbers provided in the Data Availability Section?	Yes	Data Availability Section
Were **human clinical and genomic datasets** deposited in a public access-controlled repository in accordance to ethical obligations to the patients and to the applicable consent agreement?	Not Applicable	
Are **computational models** that are central and integral to a study available without restrictions in a machine-readable form? Were the relevant accession numbers or links provided?	Not Applicable	
If publicly available data were reused, provide the respective **data citations in the reference list**.	Not Applicable	
